# Vertical stress and stability of interburden over an abandoned pillar working before upward mining: a case study

**DOI:** 10.1098/rsos.180346

**Published:** 2018-08-08

**Authors:** Jinwen Bai, Guorui Feng, Shanyong Wang, Tingye Qi, Jian Yang, Jun Guo, Zhen Li, Xianjie Du, Zehua Wang, Yunlou Du, Yujiang Zhang

**Affiliations:** 1 College of Mining Technology, Taiyuan University of Technology, Taiyuan, Shanxi 030024, People's Republic of China; 2 Institute of Mining Technology, Taiyuan University of Technology, Taiyuan, Shanxi 030024, People's Republic of China; 3 Research Center of Green Mining Engineering Technology in Shanxi Province, Taiyuan, Shanxi 030024, People's Republic of China; 4 State Key Laboratory of Mining Disaster Prevention and Control Co-founded by Shandong Province and the Ministry of Science and Technology, Shandong University of Science and Technology, Qingdao, Shandong 266590, People's Republic of China; 5 ARC Centre of Excellence for Geotechnical Science and Engineering, Civil, Surveying and Environmental Engineering, The University of Newcastle, Callaghan, NSW 2308, Australia; 6 Department of Mining Engineering, West Virginia University, Morgantown, WV 26508, USA

**Keywords:** vertical stress, stability, interburden, abandoned mining zones, residual coal pillar, upward mining

## Abstract

Upward mining of the residual coal seam over an abandoned pillar working is one of the effective measures to alleviate the contradiction between limited resources and increased consumption. Interburden stability over an abandoned pillar working plays a significant role in guaranteeing the safety of upward mining; however, it has not yet been extensively studied and understood. In this study, the vertical stress of the interburden over an abandoned pillar working was first investigated. The mechanical model of the interburden was established and the damage conditions were analysed. Then, the stability of the interburden over 38502 abandoned workings in Baijiazhuang coal mine was determined by mechanical analysis and field monitoring. The results show that: (i) Vertical stress of the interburden over abandoned mining zones is clearly lower than the initial stress, indicating the existence of a de-stressed effect. Moreover, vertical stress of the interburden over residual coal pillars is greater than the initial stress, which is the evidence of a stress concentration effect. (ii) The interburden over an abandoned pillar working should be regarded as an elastic rectangular plate supported by generalized Kelvin bodies in mechanical modelling. (iii) The interburden over abandoned mining zones may experience two damage stages. In the first stage, initial plastic damage appears at the central region of interburden. In the second stage, the plastic damage evolves from the central point to the surrounding areas. (iv) The mechanical analysis and field monitoring both indicate the initial damage occurred at the central region over 38502 abandoned workings in Baijiazhuang coal mine before upward mining. Related rock control measures should be implemented in that region to guarantee the safe mining of the residual coal seam.

## 1. Introduction

Coal is one of the most important resources in many countries [1], and it constituted more than 62% of China's energy in 2016 [2]. Large-scale intensive and long-term coal mining activities not only greatly promoted China's economic development, but also intensified the contradiction between limited resources and increased consumption [3].

Excavation of residual coal resources is an effective measure to alleviate the contradiction, which can improve the recovery rate and promote sustainable development [4]. Practices of residual coal pillar extraction have been conducted in many countries including UK [5], South Africa [6,7], Iran [8], India [9–13], USA [14] and China [15]. Residual coal resources under railways, buildings and water bodies were excavated with solid or paste backfilling mining methods [16–18]. Top coal caving technology was determined for re-mining thick residual coal resources [19,20]. Short-wall mining technology was proposed to recover the residual block/boundary coal resources [21–23]. The high-wall mining system was suggested to recycle the residual end-wall resources at open pit coal mines [24,25]. Moreover, residual island coal resources were also encouraged to be mined [26–29]. The aforementioned studies mainly focused on residual coal resources with irregular shapes or isolated states, which greatly promoted the development of theories and technologies in re-mining.

The residual coal seam over abandoned workings is also a typical resource, which is distributed in many coalfields across China [30]. It has considerable reserves and enormous excavation potential. The upward mining method is generally acknowledged as a reasonable way to recover the residual coal seam over abandoned workings [31,32]. The thickness of the interburden, height of the lower coal seam, lithology of the interburden and mining interval time greatly influence the feasibility of upward mining [33–35]. Deformation and failure of the interburden had also been given much attention with similar material simulation tests, numerical modelling, theoretical analysis and field measurements. Formation and distribution of interburden fractures induced by upward mining were explored with simulated material models [36]. Time-domain characteristics of interburden failure over an abandoned longwall working were investigated with field measurements [37]. Movement of the interburden in upward mining was analysed with numerical modelling [38,39]. The damage zone and the interburden structure in upward mining were obtained based on theoretical analysis [40–42].

The studies mentioned above provided valuable and meaningful references for the safe mining of the residual coal seam over abandoned workings, which were mainly focused on the interburden stability over abandoned longwall workings. However, several small-scale coal mines adopted partial excavation methods (such as the room and pillar mining method, bord and pillar mining method, short-wall pillar mining method, high-wall pillar mining method as well as strip pillar mining method) to ensure output and benefits, which resulted in large amounts of residual coal pillars being left between adjacent abandoned workings to support the weight of overburden strata [43–49]. In general, residual coal pillars in abandoned workings do not exist individually, but are distributed in groups [44,45]. The load-bearing stress of residual coal pillars may become concentrated after partial extraction. Furthermore, huge amounts of energy were accumulated in residual coal pillars, which may exacerbate the gradual deterioration and failure of the interburden [50]. The stability of the interburden over an abandoned pillar working plays a significant role in guaranteeing the safety of upward mining; however, it has not yet been extensively studied and understood. Vertical stress is closely related to the stability of the interburden, which controls damage evolution. Therefore, it is necessary to investigate the vertical stress and stability of the interburden above abandoned pillar workings comprehensively before upward mining.

In this study, the FLAC^3D^ software was employed to investigate the vertical stress of the interburden over an abandoned pillar working. Then, a mechanical model of the interburden was established and the damage conditions were analysed. In addition, the stability of the interburden over 38502 abandoned workings in Baijiazhuang coal mine was determined by mechanical analysis and field monitoring.

## 2. Geological description of the study mine

**Figure 1.**
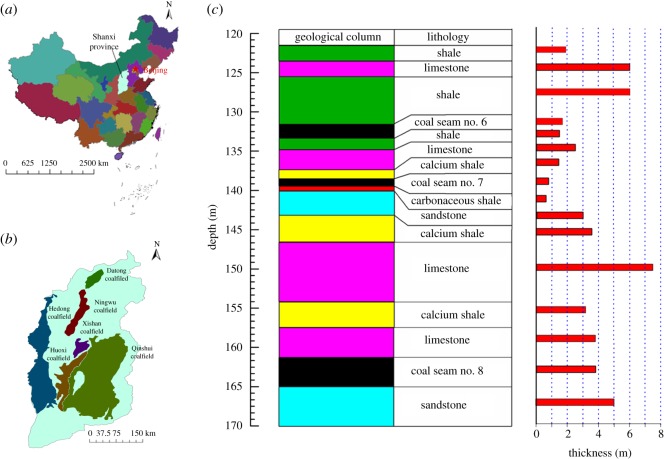
Location of Baijiazhuang coal mine and geological column of the target coal seams and rock stratas. (*a*) Shanxi Province of China; (*b*) Xishan coalfield in Shanxi Province and (*c*) typical geological column of target coal seams and rock stratas in Baijiazhuang coal mine.

As shown in figure 1, Baijiazhuang coal mine is located in Xishan coalfield of Shanxi province, China. It is well known for resource shortages, which are urgently to be addressed in order to guarantee stable output, improve productivity and promote economic benefits. The length of the mining area in Baijiazhuang mine is 4.5 km and the width is 3.2 km. The acreage of mineral zones is approximately 16.20 km^2^.

There are seven mineable seams in Baijiazhuang coal mine. Coal seams No. 6 and No. 8 are selected for this case study. The average thickness of coal seam No. 8 is 3.8 m, while it is 1.7 m for coal seam No. 6. Coal seams No. 6 and No. 8 have a dip angle of 0°, with a relatively stable and simple geological structure. The average thickness of the interburden between coal seams No. 6 and No. 8 is 27.9 m. The typical geological column of target coal seams and rock strata in Baijiazhuang coal mine is illustrated in figure 1*c*.

Coal seam No. 8 was mined prior to coal seam No. 6 with the partial excavation method in the 1990s to reap the short-term benefits. Upward mining of residual coal seam No. 6 over the abandoned pillar working has become a focus in recent years due to the lack of coal resources. However, it is restricted by the stability of residual coal pillars and the interburden, which are closely related to mining feasibility and safety. Before upward mining, the safety factor was firstly considered to evaluate the stability of the residual coal pillar, which is defined as the ratio of the coal pillar's compressive strength to the bearing load.

Generally, tributary area theory was applied to estimate the average vertical stress on coal pillars, which assumed that one pillar bears the complete weight of the ground column located above its section and above half the area between the considered pillar and the adjacent ones [51]. The bearing load of the residual coal pillar is calculated with the following equation:

2.1 $$\sigma_{p}=\rho gH_{0}\frac{{\left(a+b\right)}^{2}}{a^{2}},$$

where *σ*_p_ is the bearing load of the residual coal pillar; *ρ* is the average density of the overburden; *g* is the gravitational acceleration; *H*_0_ is the thickness of the overburden; and *a* and *b* are the width of the residual coal pillar and the abandoned mining zone, respectively.

Moreover, the strength of the residual coal pillar was determined by Bieniawski based on a great number of field tests in South Africa [52]. It is empirically expressed by the following equation and has been widely applied in a range of international settings.

2.2 $$\sigma_{s}=\sigma_{m}\left(0.64+0.36\frac{a}{H}\right),$$

where *σ*_s_ is the ultimate strength of the residual coal pillar, *σ*_m_ is the uniaxial compressive strength for the cubic specimen and *H* is the height of the residual coal pillar.

As a result, the safety factor (*f*) of the residual coal pillar can be estimated by the following equation. It is recommended that *f* > 1.3 for a stable pillar.

2.3 $$f=\frac{\sigma_{s}}{\sigma_{p}}=\frac{\sigma_{m}(0.64+0.36(a/H))}{\rho gH_{0}({\left(a+b\right)}^{2}/a^{2})}.$$

According to the geological and mining condition of the 38502 pillar working, the following parameters could be obtained: *ρ* = 1.13 g cm^−3^, *g *= 9.8 N kg^−1^, *H*_0_ = 164.4 m, *a* = 8 m, *b* = 24 m, *σ*_m_ = 29.79 MPa and *H* = 3.8 m. Therefore, the safety factor of the residual coal pillar in the 38502 pillar workings is determined as follows:

$$f=\frac{\sigma_{s}}{\sigma_{p}}=1.429>1.3.$$

**Figure 2.**
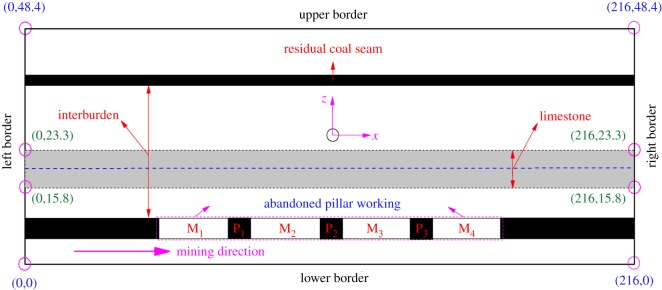
Distribution of the 38502 abandoned pillar working and the residual coal seam, in which limestone with an average thickness of 7.5 m was selected as the target strata to be investigated.

Clearly, the residual coal pillars in the 38502 pillar workings before upward mining were stable. Therefore, the stability of the interburden should be considered emphatically. In this paper, the vertical stress and stability of the interburden over the 38502 abandoned pillar working were analysed as a case study. The 38502 working was exploited with the pillar excavation method. As presented in figure 2, it consisted of four abandoned mining zones, which were named M_1_, M_2_, M_3_ and M_4_. Three horizontal residual coal pillars were left to support the overburden above the 38502 working, which were named P_1_, P_2_ and P_3_. The widths of the abandoned mining zone (M_1_, M_2_, M_3_ and M_4_) and the residual coal pillar (P_1_, P_2_ and P_3_) are 24 m and 8 m, respectively. The mining direction was from M_1_ to M_4_ successively. The immediate roof of the 38502 working is composed of limestone with an average thickness of 3.8 m, and its immediate floor is composed of sandstone with an average thickness of 5.0 m.

## 3. Vertical stress of interburden

Itasca's FLAC^3D^ software was employed to investigate the vertical stress distribution of the interburden. FLAC^3D^ is one of the most significant numerical software programs [53] and is widely used by geotechnical engineers for conducting rock mechanics calculations and solving nonlinear large deformation problems in underground excavations [54,55].

The failure criterion for numerical analysis in this investigation is the Mohr–Coulomb failure model, which was used widely in many previous studies [56,57].

The following steps are included in the numerical simulation process: (1) importing the geometric model and boundary conditions; (2) setting the geotechnical parameters and constitutive model; (3) calculating the compensated compressive vertical stress on the top of the numerical model; (4) conducting the initial balance induced by gravity; (5) simulating the excavation of M_1_; (6) outputting the vertical stress value when step (5) reaches an equilibrium state and (7) repeating the excavation simulations for M_2_, M_3_ and M_4_.

### 3.1. Numerical model scheme

#### 3.1.1. Geometric parameters and boundary conditions

**Figure 3.**
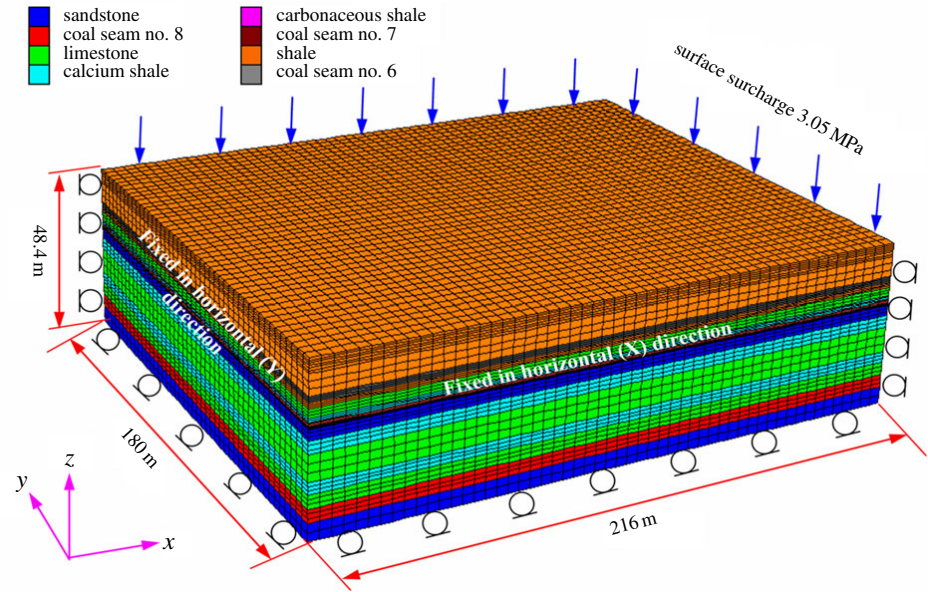
Geometric parameter and boundary conditions of the numerical model.

The numerical model is established according to the geological conditions of Baijiazhuang coal mine. Figure 3 shows the geometric parameters and boundary conditions of the three-dimensional model. Strike length, dip width and height of the numerical model are 216 m, 180 m and 48.4 m, respectively. The numerical model is composed of 116 380 nodes and 109 350 units, which mainly contains coal seam No. 6, coal seam No. 8 and the interburden strata.

Side displacements of the numerical model are fixed along the *X-* and *Y-*directions, while the bottom displacements are fixed along the *Z-*direction. The fixed boundary means that the grids in the face can slide in the planes but cannot move perpendicularly to the planes. Because the initial stress loaded on the top is equal to the overburden weight with a depth of 164.4 m, an equivalent compensated vertical stress of 3.12 MPa is applied in the negative *z*-direction to represent the *in situ* stress. Moreover, border coal pillars are designed around the sides to avoid any mechanical boundary effect. The width of the border coal pillars is 48 m along the strike direction, while it is 20 m along the dip direction.

#### 3.1.2. Monitoring lines

Studies from similar simulation tests and site investigations demonstrated that: the thick and hard strata, termed as ‘dominant strata’, plays a controlling role in bearing the overburden and guaranteeing the safety of upward mining [30,58]. Based on the stratigraphic and geomechanical information collected at Baijiazhuang coal mine, limestone with an average thickness of 7.5 m can be regarded as the dominant strata. Therefore, it is identified as the targeted monitoring strata.

**Figure 4.**
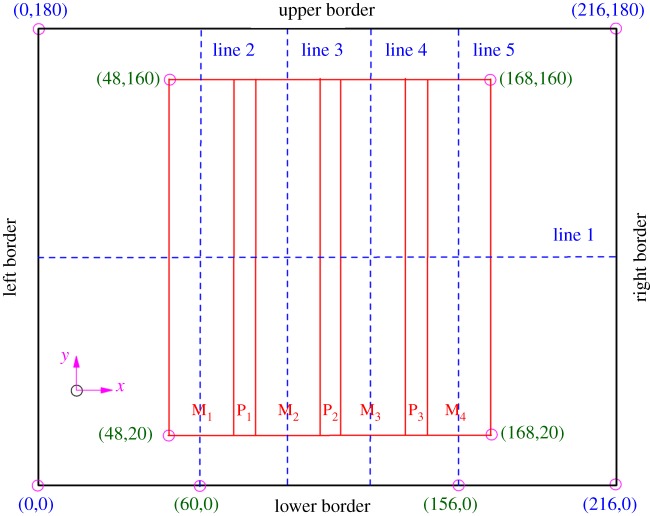
Layout of monitoring lines in the target strata.

As shown in figure 4, there are five monitoring lines in the targeted strata. Line 1 is located horizontally along the strike direction, which is coincident with the central line along the dip direction (*z* = 90 m). Line 2, line 3, line 4 and line 5 are arranged along the *z*-direction in the region from *x *= 60 m to *x *= 156 m. These monitoring lines are equidistant with a spacing of 32 m, which are coincident with the centreline of M_1_, M_2_, M_3_ and M_4_, respectively.

#### 3.1.3. Geotechnical parameters

Importing the geotechnical parameter is very important for numerical modelling. Therefore, the physical and mechanical properties of rock/coal must be assessed properly, which are generally determined by laboratory testing. According to the requirements of the Mohr–Coulomb criterion, the density, Poisson's ratio, elastic modulus, bulk modulus, shear modulus, friction angle, cohesion and tensile strength are indispensable in FLAC^3D^ numerical modelling. Exploration drilling was conducted in Baijiazhuang coal mine at five different zones in order to obtain the standard samples and test the geotechnical parameters. Then, the uniaxial compressive tests were carried out to obtain the compressive strength, Poisson's ratio and elastic modulus. The Brazilian test was conducted to investigate the tensile strength. The internal friction angle and cohesion were acquired by shear tests. Moreover, the bulk modulus and shear modulus can be calculated by the following formula [59]:

3.1 $$\left\{ \begin{array}{l} K=\frac{E}{3(1-2\text{v})}\\ G=\frac{E}{2(1+\text{v})}, \end{array}\right.$$

where *K* is the bulk modulus, *G* is the shear modulus, *E* is the elastic modulus and *v* is the Poisson's ratio.

The geotechnical parameters for the Mohr–Coulomb failure model used in the numerical simulation are given in table 1.

**Table 1.** Geotechnical parameters of rock and coal in the model.

lithology	density (g cm^−3^)	Poisson's ratio	elastic modulus (GPa)	bulk modulus (GPa)	shear modulus (GPa)	friction angle (°)	cohesion (MPa)	tensile strength (MPa)
sandstone	2.61	0.14	1.95	0.90	0.86	40.9	5.54	2.6
limestone	2.71	0.22	5.95	3.54	2.44	35.0	6.40	4.0
calcium shale	2.72	0.18	4.86	2.53	2.06	41.5	6.61	2.1
shale	2.16	0.22	0.72	0.43	0.30	33.2	2.12	0.9
carbonaceous shale	2.52	0.13	1.54	0.69	0.68	39.8	2.77	1.1
coal seam No. 6	1.31	0.32	0.21	0.19	0.08	34.2	0.58	0.4
coal seam No. 7	1.34	0.32	0.27	0.25	0.10	30.8	0.67	0.4
coal seam No. 8	1.13	0.31	0.21	0.28	0.12	34.6	0.63	0.5

### 3.2. Vertical stress of interburden

Vertical stress evolution along the strike length and dip width in the formation of the abandoned working is explored emphatically.

#### 3.2.1. Vertical stress along strike length

**Figure 5.**
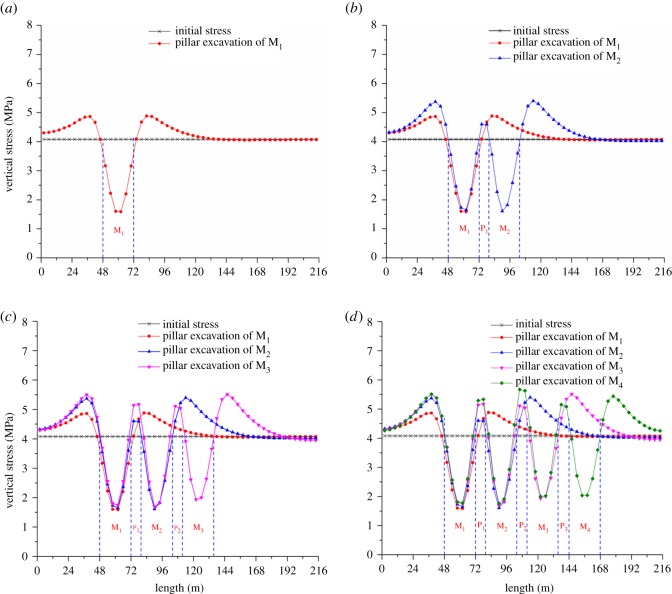
Vertical stress evolution along the strike length. (*a*) Vertical stress distribution after pillar excavation of M_1_, (*b*) vertical stress distribution after pillar excavation of M_2_, (*c*) vertical stress distribution after pillar excavation of M_3_ and (*d*) vertical stress distribution after pillar excavation of M_4_.

The stress distributions along line 1 after pillar excavation of M_1_, M_2_, M_3_ and M_4_ are shown in figure 5. The ordinate value indicates the vertical stress. The initial vertical stress induced by gravity is 4.08 MPa. It indicates the stress concentration when the vertical stress is more than 4.08 MPa; however, relief effects occur when the vertical stress is less than 4.08 MPa. The abscissa value represents the strike length of the numerical model.

After exaction of M_1_, there is a total de-stressed belt within the mining zone, which exhibits a ‘V’ shape. As shown in figure 5*a*, the vertical stress drops significantly in the area ranging from 48 to 60 m. As the horizontal distance to line 2 expands, the decreasing magnitude of vertical stress weakens gradually. The vertical stress distribution shows a symmetrical trend in the area ranging from 60 to 72 m. The minimum value appears in the middle position of M_1_. The stress concentration area with a width of 48 m also forms on both coal sides of M_1_. On the left side, the extent of stress concentration increases gradually from 0 to 36 m, while it drops significantly from 38 to 48 m. On the right side, the extent of stress concentration increases sharply from 72 to 82 m, while it drops slowly from 82 to 120 m. The maximum value is approximately 4.88 MPa.

As shown in figure 5*b*, the distribution shape of vertical stress is much like a ‘W’ after pillar excavation of M_2_. The vertical stress of P_1_ ranging from 72 to 80 m is still greater than the initial stress. In the M_2_ range from 80 to 104 m, a total de-stressed belt is also formed and the evolution pattern is similar to that in the area of M_1_. The degrees of stress concentration in the left side of M_1_ (ranging from 0 to 48 m) and the right side of M_2_ (ranging from 104 to 152 m) are both increased. The stress evolution ranging from 104 to 152 m resembles the one from 72 to 120 m in figure 5*a*. The maximum value of vertical stress in this area is approximately 5.37 MPa.

The vertical stress of M_3_ and M_4_ becomes less than the initial stress after pillar excavation, as presented in figure 5*c*,*d*. The shapes of vertical stress distribution in the mining area are shown as ‘VVV’ and ‘VVVV’ after excavation of M_3_ and M_4_, respectively. Moreover, higher vertical stress is also obvious in the areas of P_3_ and P_4_. The vertical stress evolution trend is similar to that mentioned in the previous two paragraphs.

#### 3.2.2. Vertical stress along dip width

**Figure 6.**
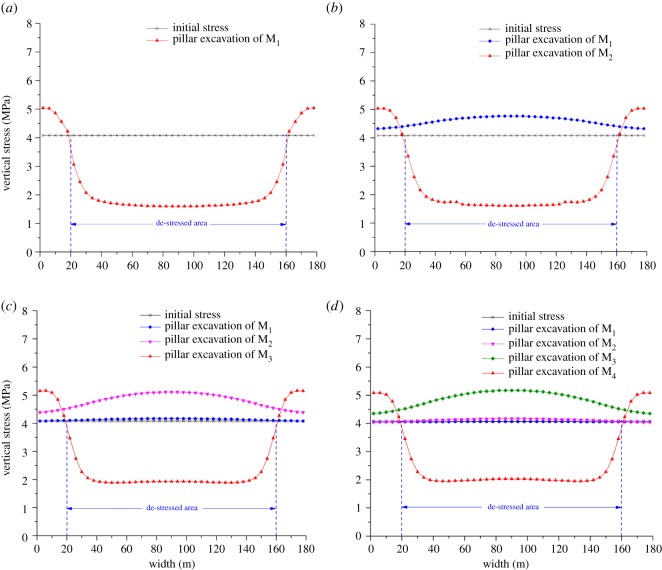
Vertical stress evolution along the dip width. (*a*) Vertical stress distribution along line 2, (*b*) vertical stress distribution along line 3, (*c*) vertical stress distribution along line 4 and (*d*) vertical stress distribution along line 5.

Figure 6 displays the vertical stress distribution along lines 2, 3, 4 and 5 after pillar excavation of M_1_, M_2_, M_3_ and M_4_, respectively. The meaning of the ordinate value is the same as that in figure 5. The abscissa value indicates the dip width of the numerical model.

For line 2, the vertical stress in the area of M_1_ is less than the initial stress, while it is more than 4.08 MPa on the sides of the upper and lower border pillars. As shown in figure 6*a*, the vertical stress distribution shape is much like a ‘U’ in M_1_. It decreases significantly in the range from 18 to 38 m, remains at a stable value of approximately 1.60 MPa in the range from 38 to 142 m and grows sharply in the range from 142 to 162 m.

The vertical stress distribution along line 3 is shown in figure 6*b*. With the effect of abutment pressure induced by pillar excavation of M_1_, the vertical stress of line 3 is more than the initial stress, which is presented in an ‘inverted parabola’ shape. The maximum value is at the middle position of line 3. After pillar excavation of M_2_, vertical stress evolution is much like that in figure 6*a* when M_1_ was excavated.

The shape of the vertical stress distribution along line 4 is also like an ‘inverted parabola’ after pillar excavation of M_1_ and M_2_. As shown in figure 6*c*, the mining influence of M_1_ is clearly weaker than that of M_2_. Moreover, vertical stress evolution along line 4 is also exhibited in a ‘U’ shape after pillar excavation of M_3_. Similarly, vertical stress concentration effects along line 5 are also generated before the excavation of M_4_. As presented in figure 6*d*, vertical stress in the range from 20 to 160 m is also less than the initial stress after pillar excavation of M_4_.

#### 3.2.3. Vertical stress distribution contour of interburden

**Figure 7.**
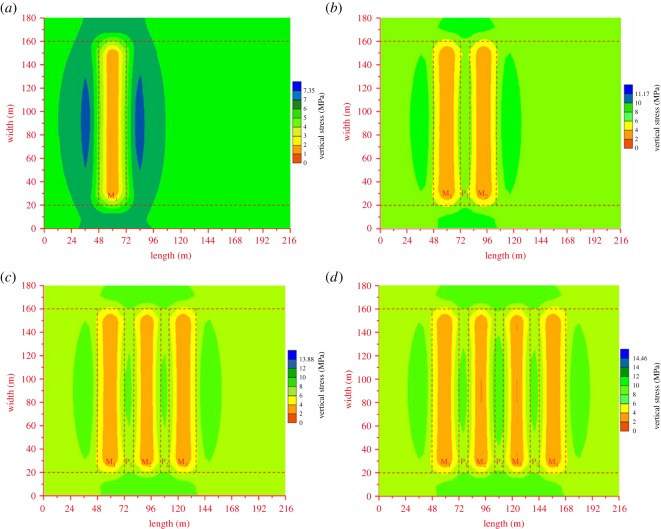
Vertical stress distribution contour after pillar excavation of coal seam No. 8. (*a*) After pillar excavation of M_1_, (*b*) after pillar excavation of M_2_, (*c*) after pillar excavation of M_3_ and (*d*) after pillar excavation of M_4_.

The vertical stress distribution contours of target strata are shown in figure 7. The ordinate and abscissa values reflect the dip width and strike length of the numerical model, respectively.

As shown in figure 7*a*, the de-stressed zone is distributed in the mining zone of M_1_, while the concentrated stress zone is located around M_1_. With the development of pillar excavation, the mining zones (M_2_, M_3_ and M_4_) evolve into de-stressed zones. The residual coal pillars (P_1_, P_2_, P_3_ and P_4_) convert into concentrated stress zones. Meanwhile, the concentrated stress zone can spread into the border coal pillars. The de-stressed zones and concentrated stress zones are presented alternately along the strike length of the numerical model after pillar excavation.

Above all, the numerical modelling results from qualitative and quantitative perspectives both illustrate that: the vertical stress of the interburden over abandoned mining zones is obviously less than the initial stress, which indicates the existence of a de-stressed effect. Furthermore, the vertical stress of the interburden over residual coal pillars is more than the initial stress, which is the evidence of a stress concentration effect.

## 4. Stability of interburden

Abundant original cracks are distributed in the interburden before excavation. Crack expansion and shrinkage are significantly influenced by vertical stress. Cracks expand when the vertical stress is decreased [59–60]. The instability potential of the interburden is enhanced when cracks expand to connect and form a network. Owing to the lower vertical stress, the cracks of the interburden over M_1_, M_2_, M_3_ and M_4_ expand, which will also increase the failure possibility. The stability of the interburden over the abandoned pillar working will be analysed with mechanical analysis and investigated with field monitoring.

### 4.1. Mechanical analysis of stability

#### 4.1.1. Establishment of mechanical model

In general, the strength of residual coal pillars in the abandoned working will be deteriorated with the coupled effects of creep deformation, environment weathering, mining-induced stress and other unfavourable factors. Furthermore, progressive failure may occur, which will further influence the stability of the interburden. Therefore, the rheological characteristics and properties of residual coal pillars should be considered in establishing the mechanical model of the interburden.

##### 4.1.1.1. Mechanical model of residual coal pillars

**Figure 8.**
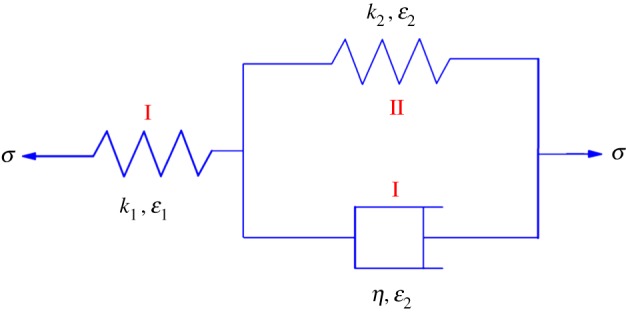
Mechanical model of a residual coal pillar.

In this paper, each residual coal pillar is regarded as a generalized Kelvin body. The mechanical model of a residual coal pillar is shown in figure 8, which is composed of an elastic unit (spring-I) and a Kelvin model (damper-I and spring-II) [60–62].

The constitutive equation of a generalized Kelvin body could be expressed as follows:

4.1 $$\frac{\eta }{k_{1}}\dot{\sigma }+\left(1+\frac{k_{2}}{k_{1}}\right)\sigma =\eta \dot{\varepsilon }+k_{2}\varepsilon ,$$

where *η* is the viscous coefficient of damper-I, *k*_1_ and *k*_2_ are the elastic stiffness of spring-I and spring-II, respectively, *ε* is the strain and *σ* is the bearing load.

##### 4.1.1.2. Mechanical model of interburden

**Figure 9.**
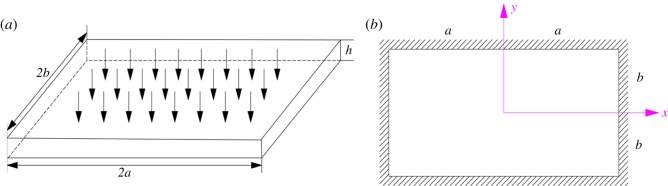
Elastic rectangular plate with four fixed sides. (*a*) The three-dimensional model of the interburden and (*b*) the two-dimensional model of the interburden.

After partial mining, the interburden is supported by residual coal pillars in the abandoned working. Owing to lateral constraints, the interburden can be considered as an elastic rectangular plate with four fixed boundaries (figure 9).

As shown in figure 9, the length of the elastic rectangular plate is 2*a*, the width is 2*b* and the thickness is *h* (*b* < *a*). The elastic modulus, Poisson's ratio and bulk density are expressed as *E*, *v* and *ρ*, respectively. The tensile strength limit is supposed as [*σ*_s_].

**Figure 10.**
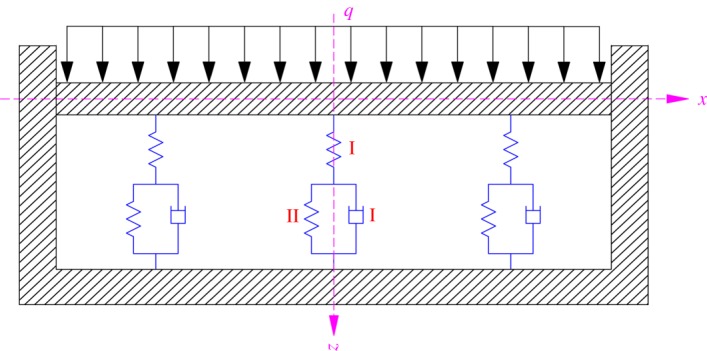
Mechanical model of the interburden over the abandoned pillar working.

Furthermore, the interburden over the abandoned pillar working can be regarded as an elastic rectangular plate supported by generalized Kelvin bodies. Figure 10 presents the mechanical model of the interburden over the abandoned pillar working. Three equidistant generalized Kelvin bodies are considered in the mechanical model. Moreover, the vertical stress above the elastic rectangular plate is assumed to be uniformly distributed load and is expressed as *q*.

According to the theories of elastic mechanics [63], the interburden subsidence over the abandoned pillar working is determined by the following equation:

4.2 $$D\nabla^{4}w+k_{1}w_{1}+k_{2}w_{2}+\eta {\dot{w}}_{2}-q=0,$$

where $D=Eh^{3}/12(1-v^{2})$ is the bending stiffness of the elastic rectangular plate, $\nabla^{4}=\partial^{4}/\partial x^{4}+2(\partial^{4}/\partial x^{2}y^{2})+\partial^{4}/\partial y^{4}$ is the bi-harmonic operator, *w* is the interburden deformation and *w*_1_ and *w*_2_ are the deformations of spring-I and spring-II, respectively.

#### 4.1.2. Mechanical solution of stability

The boundary conditions of the elastic rectangular plate before excavation are shown as follows:

4.3 $$w{|}_{x=\pm a}=0,\;w{|}_{y=\pm b}=0,\;{\frac{\partial w}{\partial x}|}_{x=\pm a}=0,\;{\frac{\partial w}{\partial y}|}_{y=\pm b}=0.$$

According to the constraint characteristics, the deflection of the elastic rectangular plate can be assumed as follows:

4.4 $$w(x,y,z)=\frac{w_{0}(t)}{a^{4}b^{4}}{\left(x^{2}-a^{2}\right)}^{2}{\left(y^{2}-b^{2}\right)}^{2},$$

where *w*_0_(*t*) = *w*(0, 0, *t*) is the maximum deflection in the centre of the interburden.

By setting $\varphi (x,y)={\left(x^{2}-a^{2}\right)}^{2}{\left(y^{2}-b^{2}\right)}^{2}/a^{4}b^{4}$, *w*(*x*, *y*, *t*) can be expressed as follows:

4.5 $$w(x,y,t)=w_{0}(t)\varphi (x,y).$$

By substituting equation (4.2) into equation (2.2), the following equation can be obtained:

4.6 $$\int_{-a}^{a}\int_{-b}^{b}\left(D\nabla^{4}w+kw+\eta \dot{w}-q\right)\varphi \text{d}x\text{d}y=0.$$

Moreover, the bending moment expressions of the elastic rectangular plate are given as follows:

4.7 $$\begin{array}{rl} M_{x} & =-D\left(\frac{\partial^{2}w}{\partial x^{2}}+v\frac{\partial^{2}w}{\partial y^{2}}\right)=-\frac{4Dw_{0}(t)}{a^{4}b^{4}}[(3x^{2}-a^{2}){\left(y^{2}-b^{2}\right)}^{2}+v{\left(x^{2}-a^{2}\right)}^{2}(3y^{2}-b^{2})]\\ \;\text{and}\;\;M_{y} & =-D\left(\frac{\partial^{2}w}{\partial y^{2}}+v\frac{\partial^{2}w}{\partial x^{2}}\right)=-\frac{4Dw_{0}(t)}{a^{4}b^{4}}[{\left(x^{2}-a^{2}\right)}^{2}{\left(3y^{2}-b^{2}\right)}^{2}+v(3x^{2}-a^{2}){\left(y^{2}-b^{2}\right)}^{2}]. \end{array}\}$$

Based on the extreme value of *M_x_*(*x*, *y*) and *M_y_*(*x*, *y*) in the range of – *a* ≤ *x* ≤ *a* and – *b* ≤ *y* ≤ *b*, |*M_x_*|_max_ and |*M_y_*|_max_ can be calculated in the coordinates of (± *a*,0) and (0,± *b*), respectively, which can be written as follows:

4.8 $$\begin{array}{rl} |M_{x}{|}_{\max} & =\frac{8Dw_{0}(t_{0})}{a^{2}}\\ \;\text{and}\;\;|M_{y}{|}_{\max} & =\frac{8Dw_{0}(t_{0})}{b^{2}}. \end{array}\}$$

Therefore, the initial plastic damage appears at the midpoint, and the damage conditions are calculated as follows:

4.9 $$\begin{array}{rl} {\sigma_{x}}_{\max} & =\frac{6{\left|M_{x}\right|}_{\max}}{h^{2}}=\frac{48Dw_{0}(t)}{a^{2}h^{2}}\geq [\sigma_{s}]\\ \;\text{and}\;\;{\sigma_{y}}_{\max} & =\frac{6{\left|M_{y}\right|}_{\max}}{h^{2}}=\frac{48Dw_{0}(t)}{b^{2}h^{2}}\geq [\sigma_{s}]. \end{array}\}$$

With the evolution of the initial damage, the fixed boundaries of the interburden are converted into hinged ones, which meet the following conditions:

4.10 $$w{|}_{x=\pm a}=0,\;w{|}_{y=\pm b}=0,\;{\frac{\partial^{2}w}{\partial x^{2}}|}_{x=\pm a}=0,\;{\frac{\partial^{2}w}{\partial y^{2}}|}_{y=\pm b}=0.$$

In this case, the deflection of the elastic rectangular plate can be supposed as follows:

4.11 $$w=w_{0}\cos\frac{\pi x}{2a}\cos\frac{\pi y}{2b}.$$

By substituting equation (4.8) into equation (2.2), the following equation can be obtained:

4.12 $$\iint (D\nabla^{4}w_{2}+k_{2}w_{2}+\eta {\dot{w}}_{2}-q)\cos\frac{\pi x}{2a}\cos\frac{\pi y}{2b}\text{d}x\text{d}y=0.$$

The bending moment expressions of the elastic rectangular plate are as follows:

4.13 $$\begin{array}{r} M_{x}=-\frac{D\pi^{2}w_{0}(t)}{4a^{2}b^{2}}(b^{2}+va^{2})\cos\frac{\pi x}{2a}\cos\frac{\pi y}{2b}\\ M_{y}=-\frac{D\pi^{2}w_{0}(t)}{4a^{2}b^{2}}(a^{2}+vb^{2})\cos\frac{\pi x}{2a}\cos\frac{\pi y}{2b}. \end{array}\}$$

Then, the maximum value of |*M_x_*|_max_ and |*M_y_*|_max_ can be obtained in the coordinate of (0,0):

4.14 $$\begin{array}{r} |M_{x}{|}_{\max}=\frac{\pi^{2}D(b^{2}+va^{2})w_{0}(t_{0})}{4a^{2}b^{2}}\\ |M_{y}{|}_{\max}=\frac{\pi^{2}D(a^{2}+vb^{2})w_{0}(t_{0})}{4a^{2}b^{2}}. \end{array}\}$$

Therefore, the second plastic damage expands from the central point of the interburden once the following condition is met:

4.15 $$\begin{array}{r} {\sigma_{x}}_{\max}=\frac{3\pi^{2}D}{2h^{2}}\left(\frac{1}{a^{2}}+\frac{v}{b^{2}}\right)w_{0}(t_{0})\geq [\sigma_{s}]\\ {\sigma_{y}}_{\max}=\frac{3\pi^{2}D}{2h^{2}}\left(\frac{v}{a^{2}}+\frac{1}{b^{2}}\right)w_{0}(t_{0})\geq [\sigma_{s}]. \end{array}\}$$

Above all, the interburden experiences two damage stages. In the first stage, the initial plastic damage appears from the midpoint, which results in a shift of the boundary condition. In the second stage, the plastic damage evolves from the central point of the interburden to the surrounding areas.

As for the 38502 abandoned pillar working in Baijiazhuang coal mine, the dominant strata is limestone with an average thickness (*h*) of 7.5 m. It is buried at a depth (*h*_0_) of 149.9 m. As shown in figure 4, the interburden over the 38502 abandoned pillar working is regarded as a rectangular plate with the size of 2*a* = 120 m and 2*b* = 140 m. The pillar mining height (*H*) is 3.8 m. Additionally, the necessary parameters can be obtained from table 1, which mainly include the following: the elastic modulus of the target interburden *E* = 5.95 GPa, the Poisson's ratio of the target interburden *v* = 0.22, the tensile strength of the target interburden [*σ*_s_] = 4 MPa and the elastic modulus of the coal pillar *E*_1_ = 0.21 GPa. Moreover, the mined-out ratio of *λ* is 80%. The *γ*_0_ and *γ* are determined to be 21 kN m^−3^ and 24 kN m^−3^, respectively.

The calculation procedure is as follows:

(1) The uniformly distributed load above the elastic rectangular plate:
  $$q=q_{0}+\gamma h=\gamma_{0}h_{0}+\gamma h=21\;\text{kN}\;{\text{m}}^{-3}\times 149.9\;\text{m}+24\;\text{kN}\;{\text{m}}^{-3}\times 7.5\;\text{m}=3.3279\;\text{MPa}.$$
(2) The bending stiffness of the elastic rectangular plate:
  $$D=\frac{Eh^{3}}{12(1-v^{2})}=\frac{5.95\times 10^{3}\times 7.5^{3}}{12(1-0.22^{2})}=2.198\times 10^{5}\;\text{MPa}\;\cdot \;{\text{m}}^{3}.$$
(3) The maximum deflection in the central position of the elastic rectangular plate:
  $$\begin{array}{rl} w_{0}(t_{0}) & =\frac{441}{128}\frac{q}{2\lambda (E_{1}/H)+9D{\left(7/a^{4}+4/a^{2}b^{2}+7/b^{4}\right)}^{2}}\\ & =\frac{441}{128}\times \frac{3.3279}{2\times 0.8\times (0.21\times 10^{3}/3.8)+9\times 2.198\times 10^{5}{\left(7/60^{4}+(4/60^{2}\times 70^{2})+7/70^{4}\right)}^{2}}\\ & =0.1297\;\text{m}. \end{array}$$
(4) When the initial plastic damage occurs at the midpoint of the elastic rectangular plate, the maximum tensile stress is calculated by formula (4.9).
  $$\begin{array}{rl} {\sigma_{x}}_{\max} & =\frac{6{\left|M_{x}\right|}_{\max}}{h^{2}}=\frac{48Dw_{0}(t_{0})}{a^{2}h^{2}}=\frac{48\times 2.198\times 10^{5}\times 0.1297}{60^{2}\times 7.5^{2}}=6.75745>[\sigma_{s}]=4.0\\ \;\text{and}\;\;{\sigma_{y}}_{\max} & =\frac{6{\left|M_{y}\right|}_{\max}}{h^{2}}=\frac{48Dw_{0}(t_{0})}{b^{2}h^{2}}=\frac{48\times 2.198\times 10^{5}\times 0.1297}{70^{2}\times 7.5^{2}}=4.96466>[\sigma_{s}]=4.0. \end{array}\}$$
(5) Apparently, the condition of $\left\{ \begin{array}{l} {\sigma_{x}}_{\max}\geq [\sigma_{s}]\\ {\sigma_{y}}_{\max}\geq [\sigma_{s}] \end{array}\right.$ is met.
When the second plastic damage expands from the central point of the interburden, the maximum tensile stress is calculated by formula (4.15).
  $$\begin{array}{rl} {\sigma_{x}}_{\max} & =\frac{3\pi^{2}D}{2h^{2}}\left(\frac{1}{a^{2}}+\frac{v}{b^{2}}\right)w_{0}(t_{0})\\ & =\frac{3\pi^{2}\times 2.198\times 10^{5}}{2\times 7.5^{2}}\times \left(\frac{1}{60^{2}}+\frac{0.22}{70^{2}}\right)\times 0.1297=2.4210<[\sigma_{s}]=4\\ {\sigma_{y}}_{\max} & =\frac{3\pi^{2}D}{2h^{2}}\left(\frac{v}{a^{2}}+\frac{1}{b^{2}}\right)w_{0}(t_{0})\\ & =\frac{3\pi^{2}\times 2.198\times 10^{5}}{2\times 7.5^{2}}\times \left(\frac{0.22}{60^{2}}+\frac{1}{70^{2}}\right)\times 0.1297=1.9897<[\sigma_{s}]=4. \end{array}\}$$

Apparently, the condition of $\left\{ \begin{array}{l} {\sigma_{x}}_{\max}\geq [\sigma_{s}]\\ {\sigma_{y}}_{\max}\geq [\sigma_{s}] \end{array}\right.$ is not met. It can be concluded that: the initial damage occurred for the interburden over the 38502 abandoned pillar working, while the later damage is not observed. Therefore, the failure occurs in the central region of the interburden over the abandoned mining zones.

### 4.2. Field investigation of stability

#### 4.2.1. Field monitoring scheme

**Figure 11.**
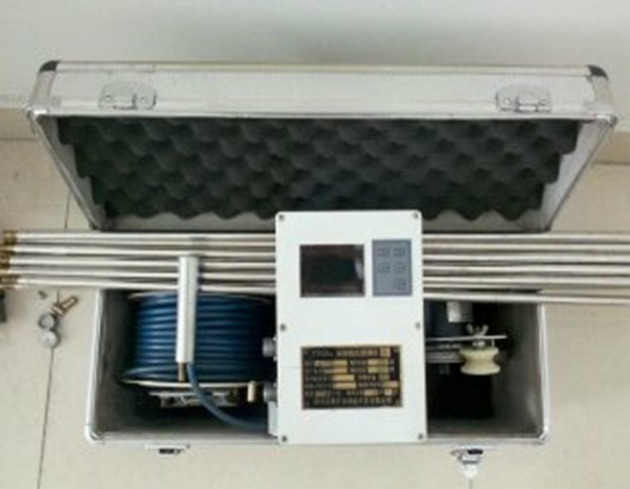
TYGD10 high-definition borehole camera detection.

To validate the analytical model and assess the stability of the interburden over the 38502 abandoned working, TYGD10 high-definition borehole camera detection technology was employed in Baijiazhuang coal mine. The system of borehole camera detection, which comprised a panoramic camera system, data lines, a data capture card, an image processor and a laptop, is an explosion-proof and portable device (figure 11). In the process of monitoring, a 360° digital and real-time image of cracking of the rock/coal mass can be recorded and stored when the camera is moved progressively down the borehole.

**Figure 12.**
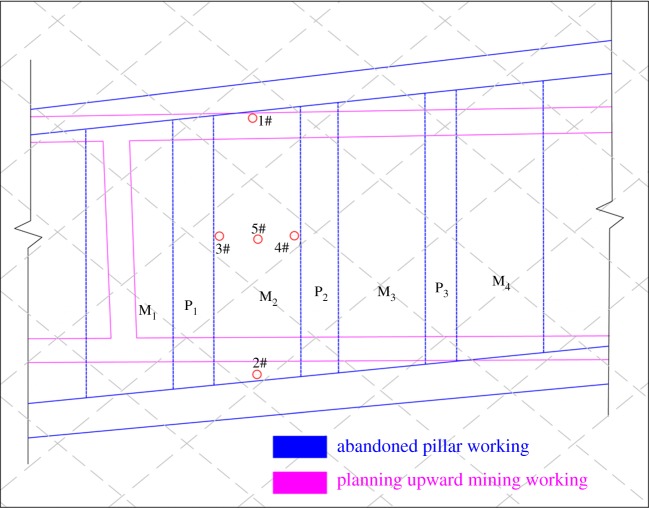
Arrangement of monitoring boreholes.

Several vertical monitoring boreholes were drilled from the immediate roof above the abandoned mining zone in 2008. Figure 12 shows the arrangement of five typical boreholes selected for case study. Boreholes 1# and 2# were drilled on the northern and southern boundaries of the M_2_ abandoned working, respectively. Boreholes 3# and 4# were drilled on the eastern and western boundaries of the 38502 abandoned working, respectively. Moreover, borehole 5# was located in the central position of the 38502 abandoned working. The depth of the boreholes was 10 m, which could reach the position of the dominant strata—7.5 m limestone. Observations of the cracking characteristics from boreholes can detect and reflect the stability of the interburden. The diameter of the borehole is 28 mm.

#### 4.2.2. Field assessment of stability

**Figure 13.**
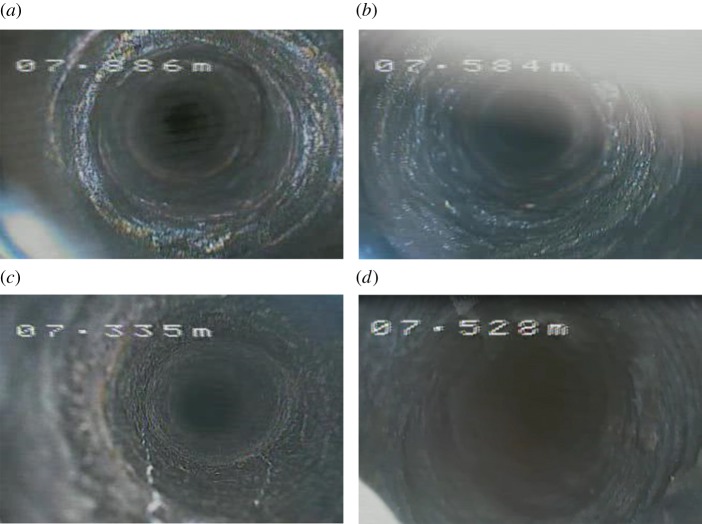
Borehole images at boundary positions. (*a*) Borehole 1#, (*b*) Borehole 2#, (*c*) Borehole 3# and (*d*) Borehole 4#.

Figure 13 presents the borehole images obtained from the boundary positions of the interburden. It could be seen from figure 13 that the boundary regions of the interburden were almost intact with some tiny annular cracks. The wall of the borehole tended to be smooth, which indicated that the stability of the interburden around the 38502 abandoned working was well.

**Figure 14.**
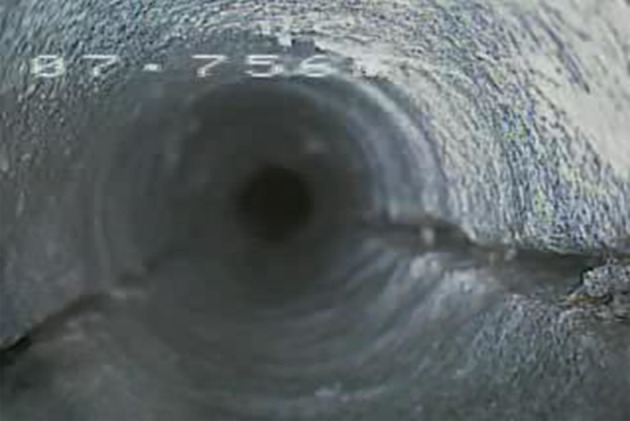
Borehole 5# image at the central position.

The central position borehole image is shown in figure 14. Apparently, large macro-cracks are observed in borehole 5#. It is the evidence of plastic damage, which indicates the failure of the interburden at the central position and is consistent with the results of the mechanical analysis.

## 5. Conclusion

In summary, the vertical stress of the interburden over an abandoned pillar working was investigated with numerical modelling. The mechanical model of the interburden was established and the damage conditions were analysed. Then, the stability of the interburden over 38502 abandoned workings in Baijiazhuang coal mine was determined by mechanical analysis and field monitoring. The following conclusions can be obtained from this study:

(1) Vertical stress of the interburden over abandoned mining zones is clearly lower than the initial stress, which indicates the existence of a de-stressed effect. Moreover, the vertical stress of the interburden over residual coal pillars is greater than the initial stress, which is the evidence of a stress concentration effect.
(2) The interburden over an abandoned pillar working should be regarded as an elastic rectangular plate supported by generalized Kelvin bodies in the mechanical model.
(3) The interburden over abandoned mining zones may experience two damage stages. In the first stage, initial plastic damage appears at the central region of the interburden. In the second stage, the plastic damage evolves from the central point to the surrounding areas.
(4) The results of mechanical analysis and field monitoring all indicate that the interburden over 38502 abandoned workings in Baijiazhuang coal mine occurred after the initial damage. In other words, the stability of the interburden at the central region is not good before upward mining. Related rock control measures should be implemented in that region to guarantee the safe mining of the residual coal seam.

## Supplementary Material

Jinwen Bai_Figures_ESM.doc

## Supplementary Material

Jinwen Bai_Figures_ESM.rar

## Supplementary Material

Jinwen Bai_Tables_ESM.doc

## Supplementary Material

Jinwen Bai_Data_ESM.rar

## Data accessibility

All data are included in the manuscript as tables and figures, which have been uploaded as the electronic supplementary material in the submission system.
