# Supplementary material for: Vertical stress and stability of interburden over an abandoned pillar working before upward mining: a case study
Source: R Soc Open Sci. 2018 Aug 8;5(8):180346. doi: 10.1098/rsos.180346 (PMC6124054; doi:10.1098/rsos.180346)
Supplement: Jinwen Bai_Figures_ESM.doc [file rsos180346supp1.doc]

| 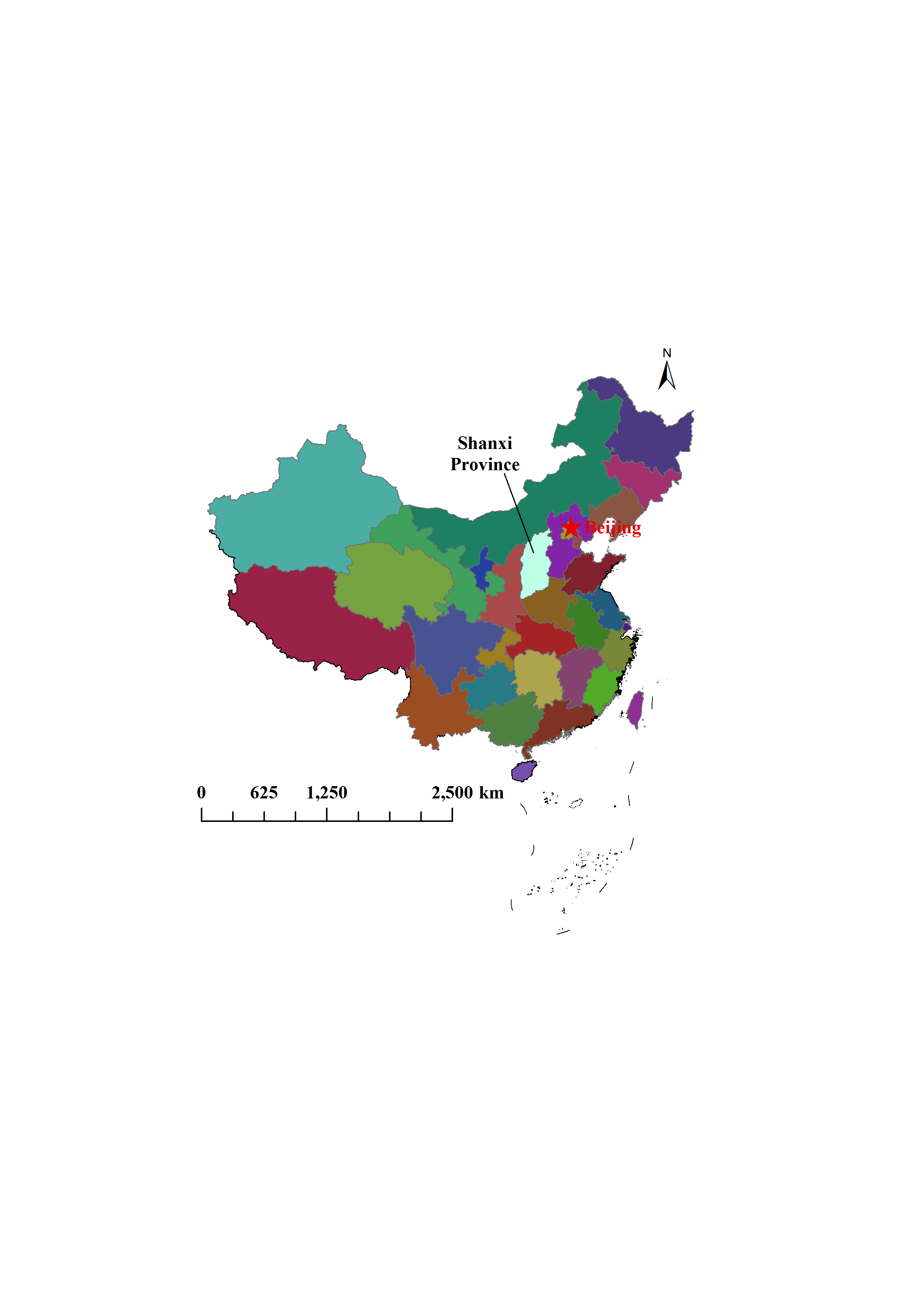  (**a**) | 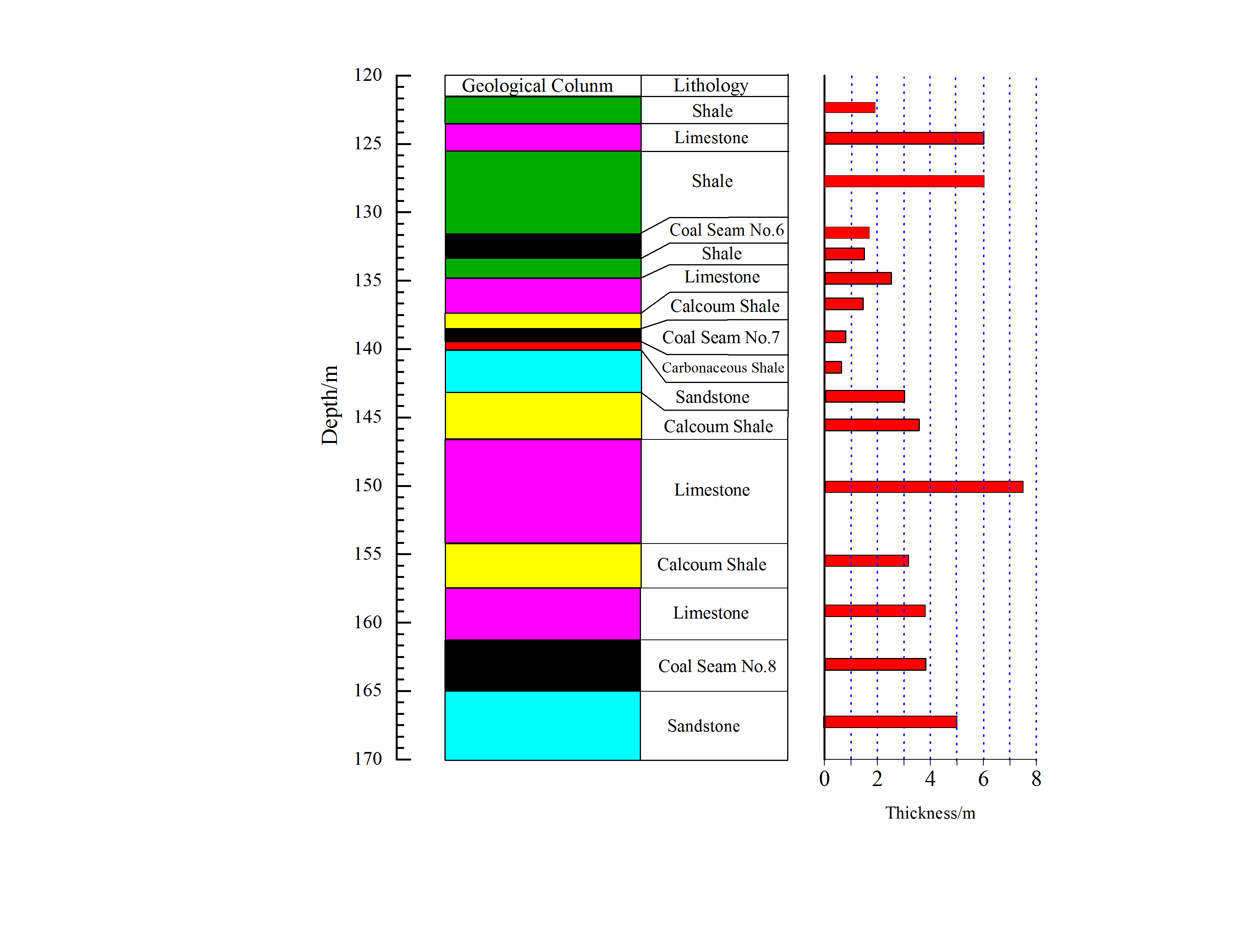  (**c**) |
| --- | --- |
| 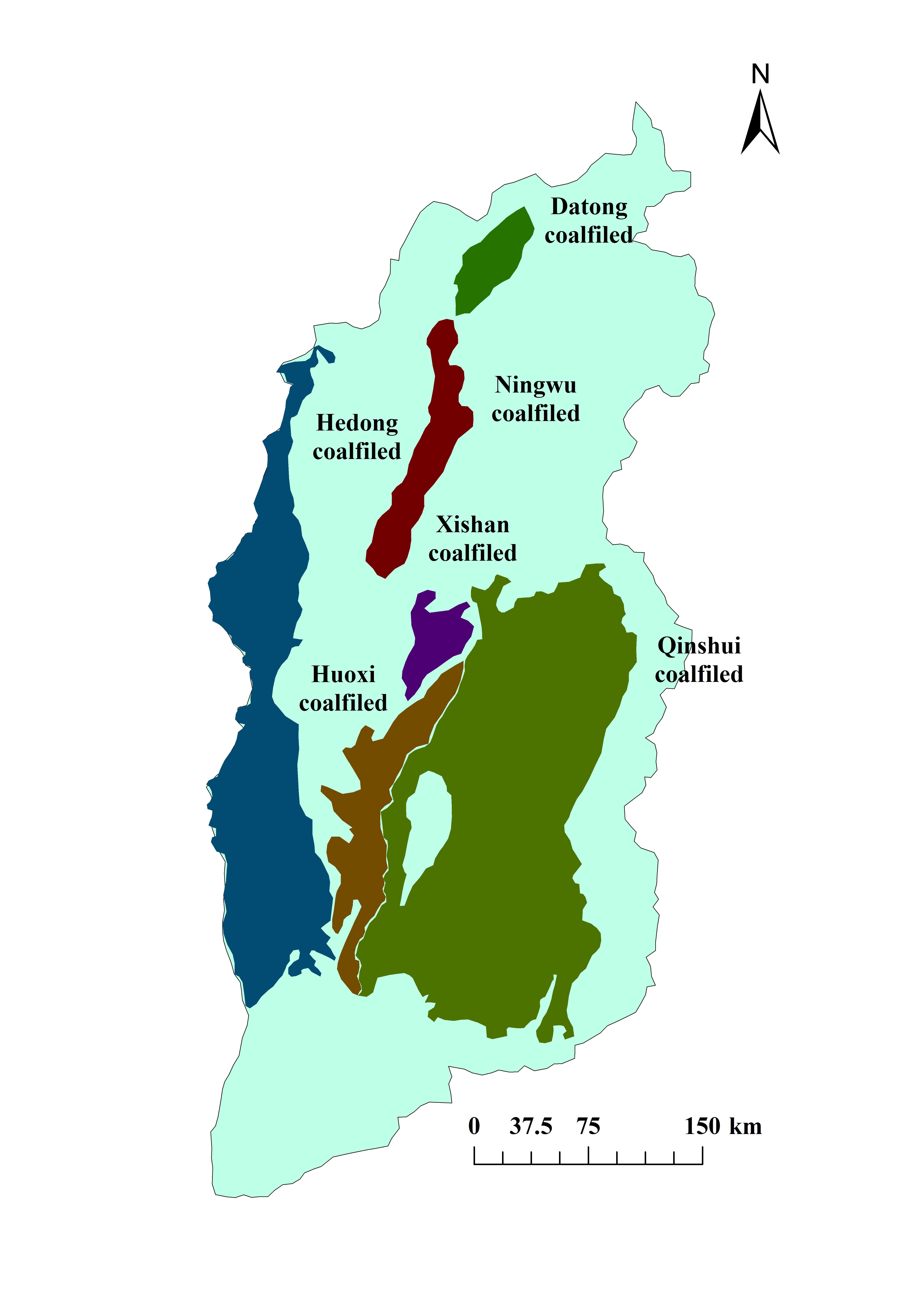  (**b**) |

**Figure 1.** Location of Baijiazhuang coal mine and geological column of target coal seam and rock stratas. **(a)** Shanxi Province of China; **(b)** Xishan coalfield in Shanxi Province; **(c)** Typical geological column of target coal seams and rock stratas in Baijiazhuang coal mine.

| 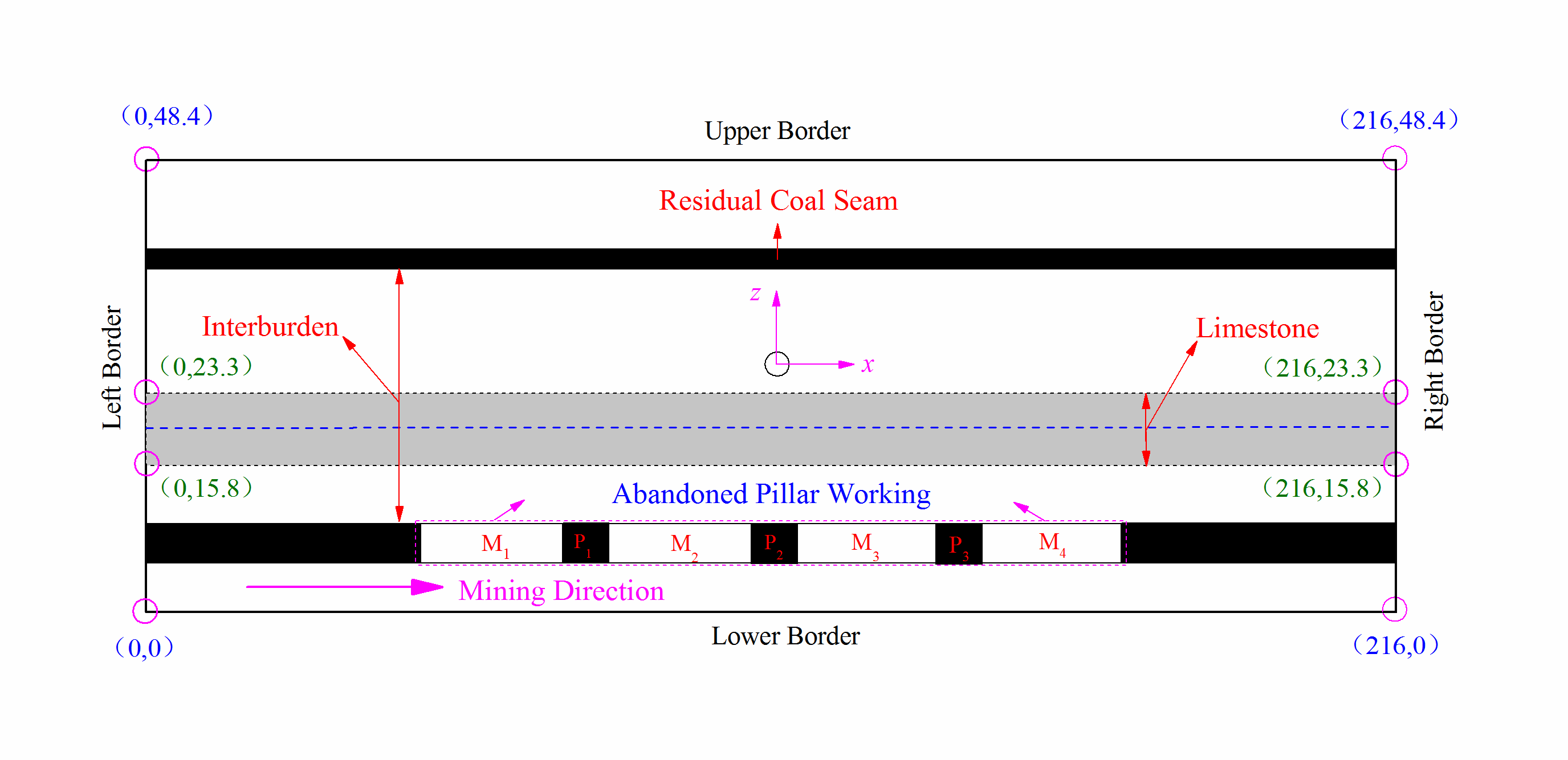 |
| --- |

**Figure 2.** Distribution of 38502 abandoned pillar working and residual coal seam, in which the limestone with 7.5m was selected as the target strata to be investigated

| **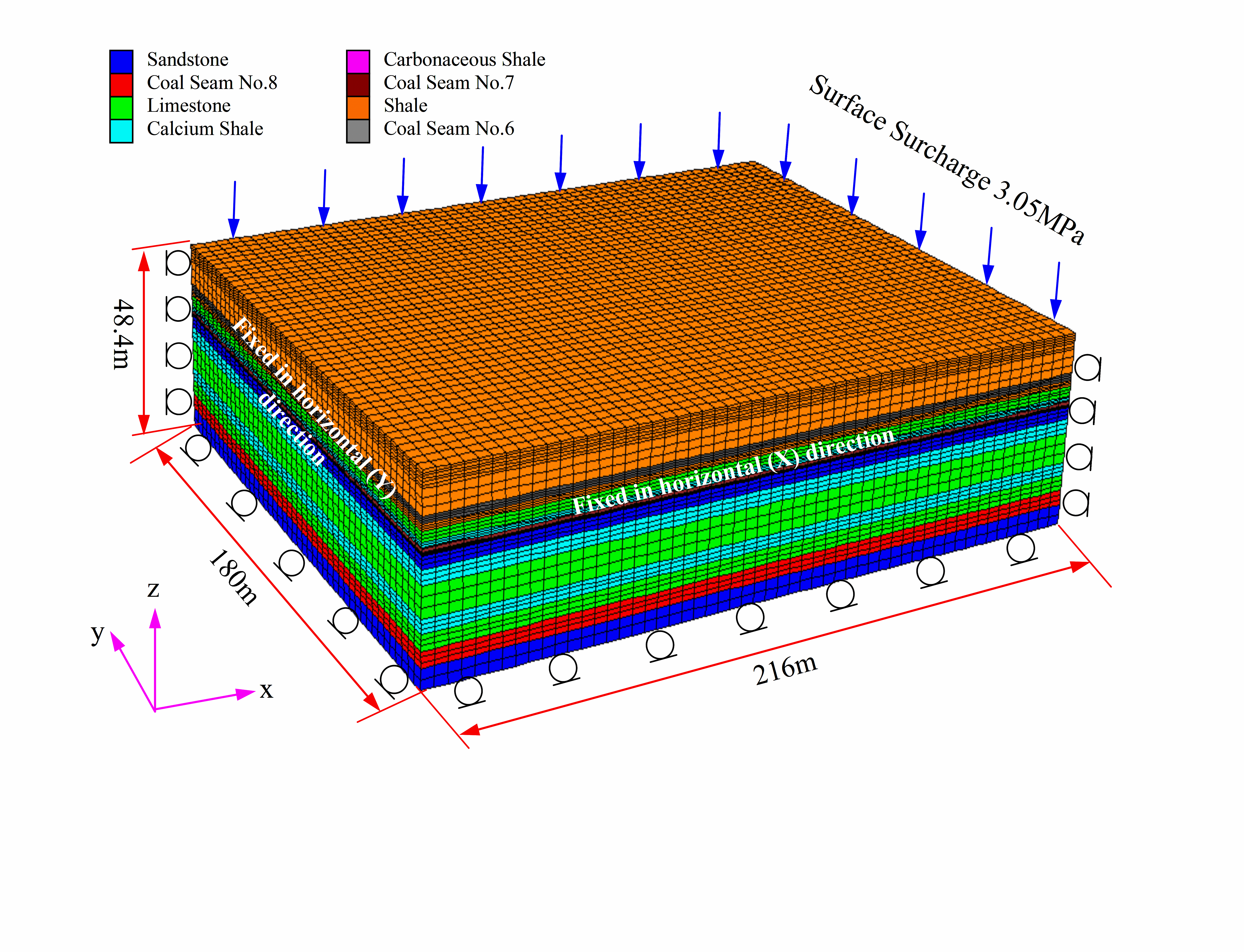** |
| --- |

**Figure 3.** Geometric parameter and boundary conditions of numerical model.

| 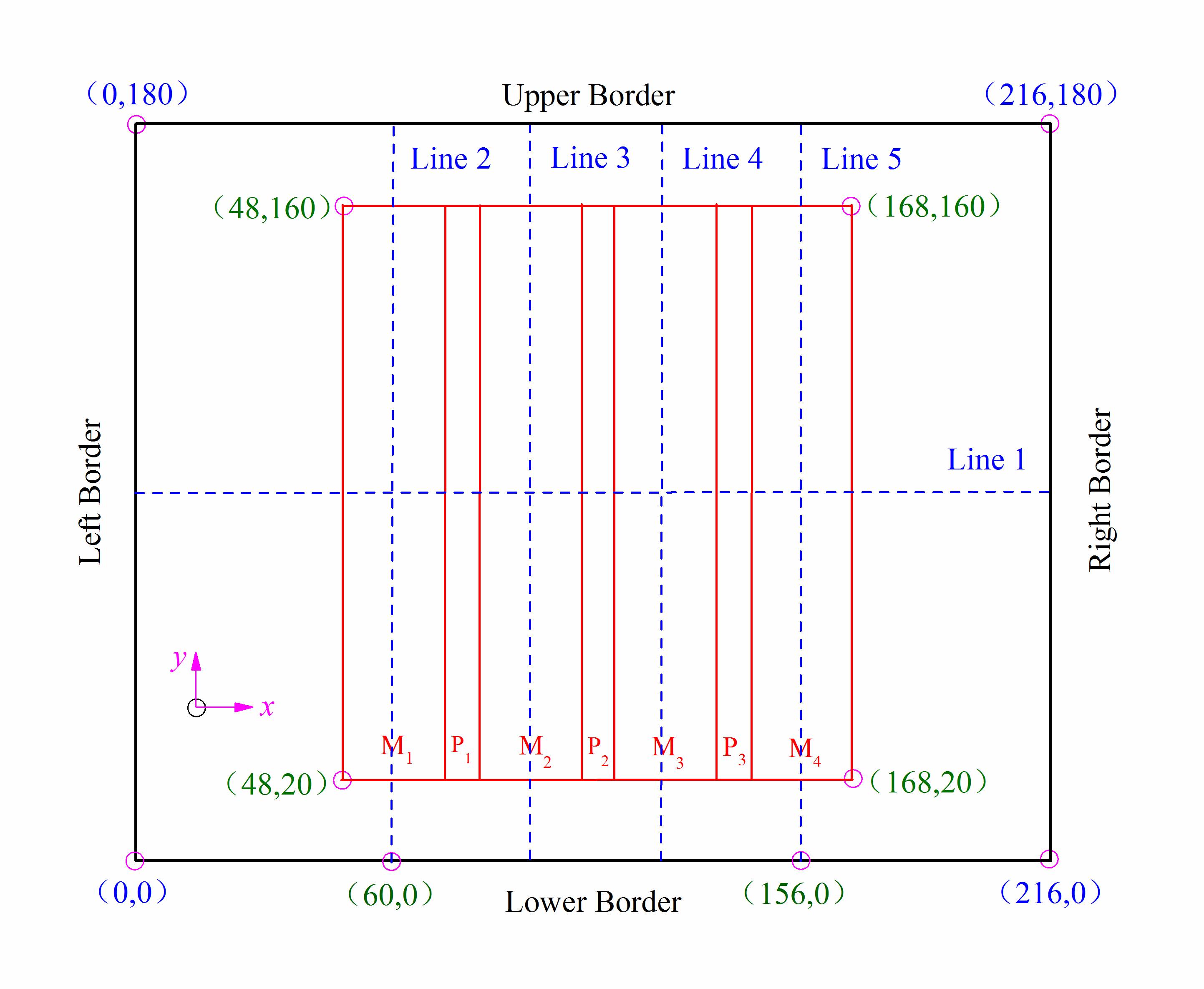 |
| --- |

**Figure 4.** Layout of monitoring lines in the target strata.

| 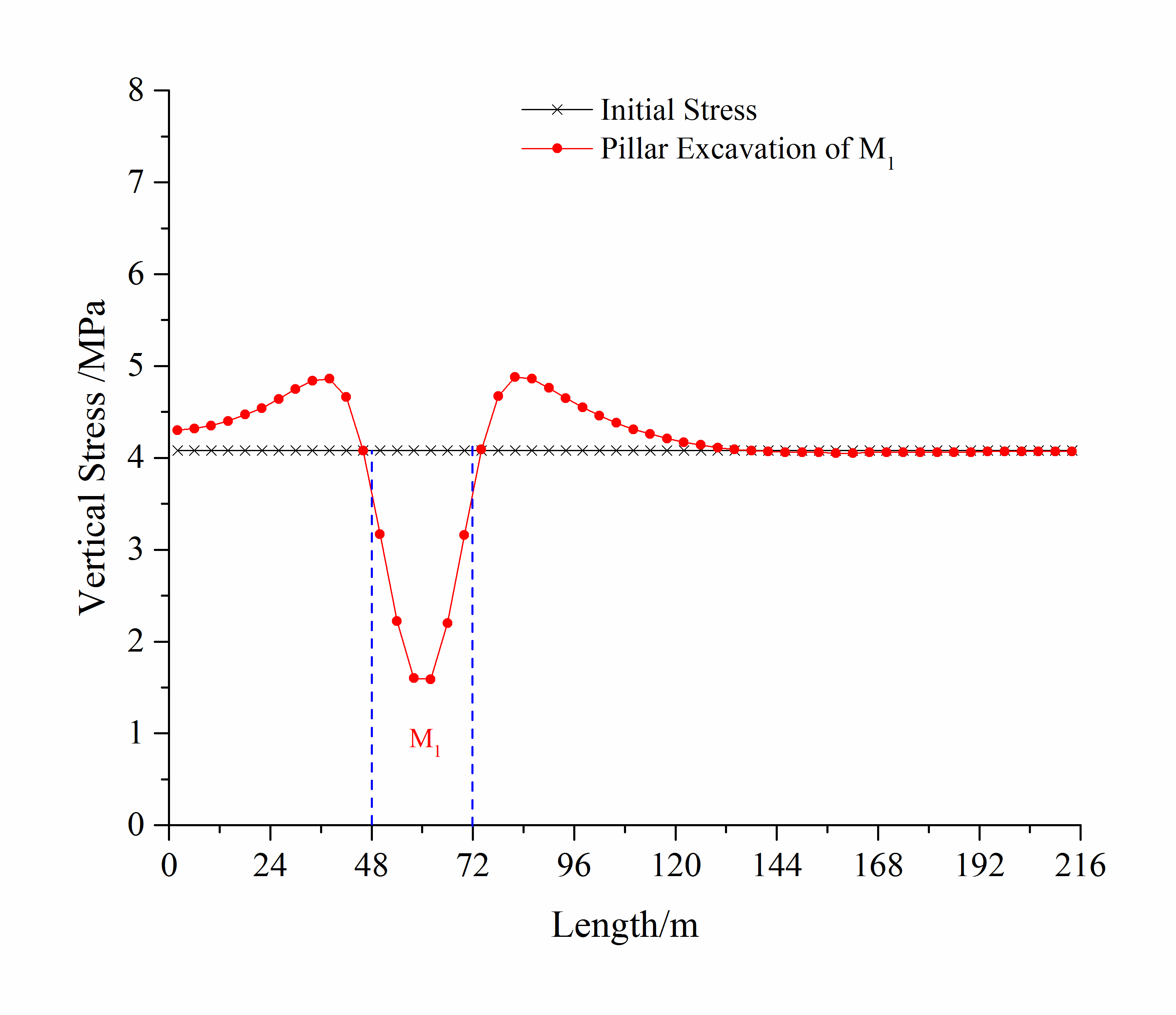  (**a**) | 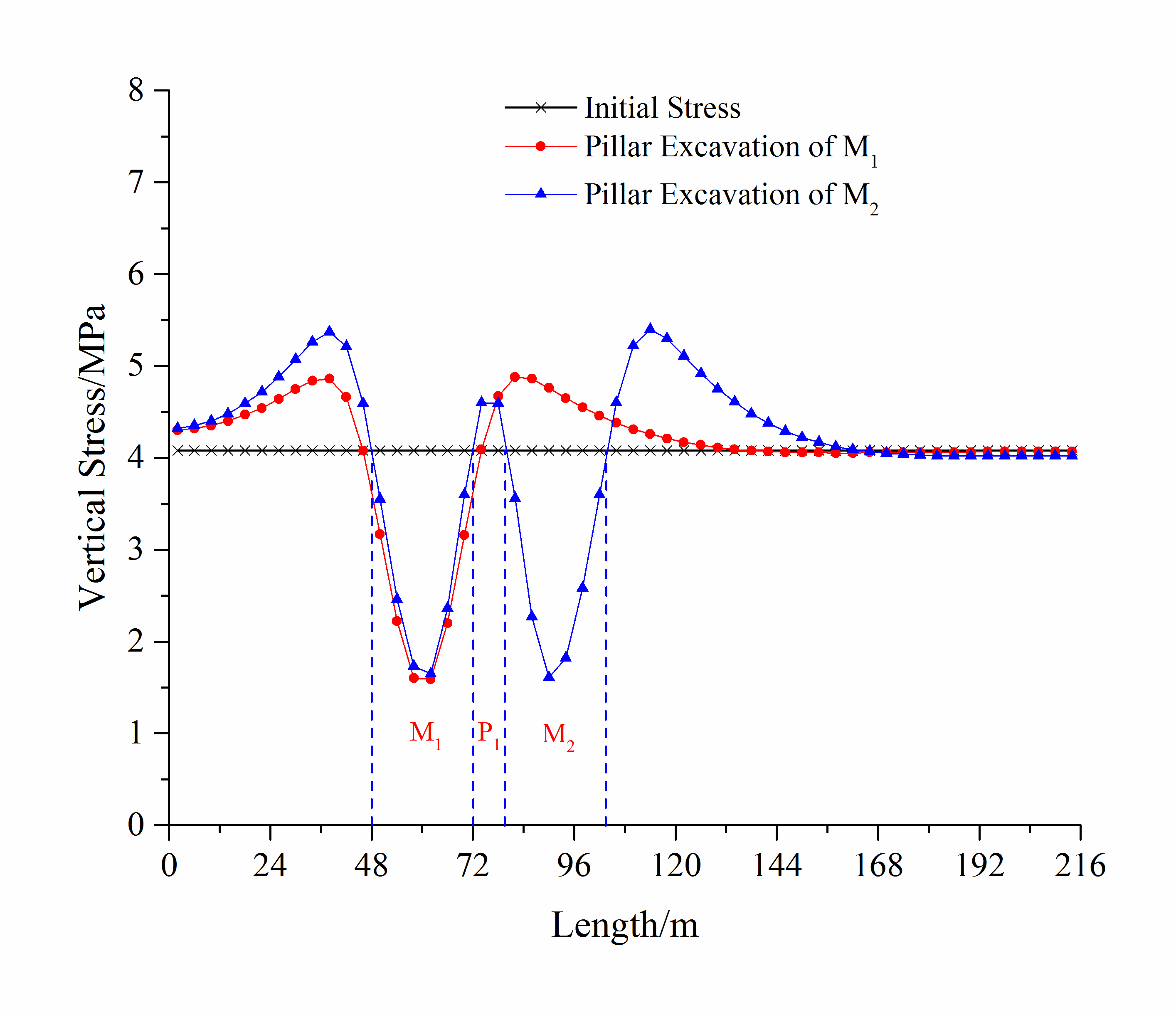  (**b**) |
| --- | --- |
| 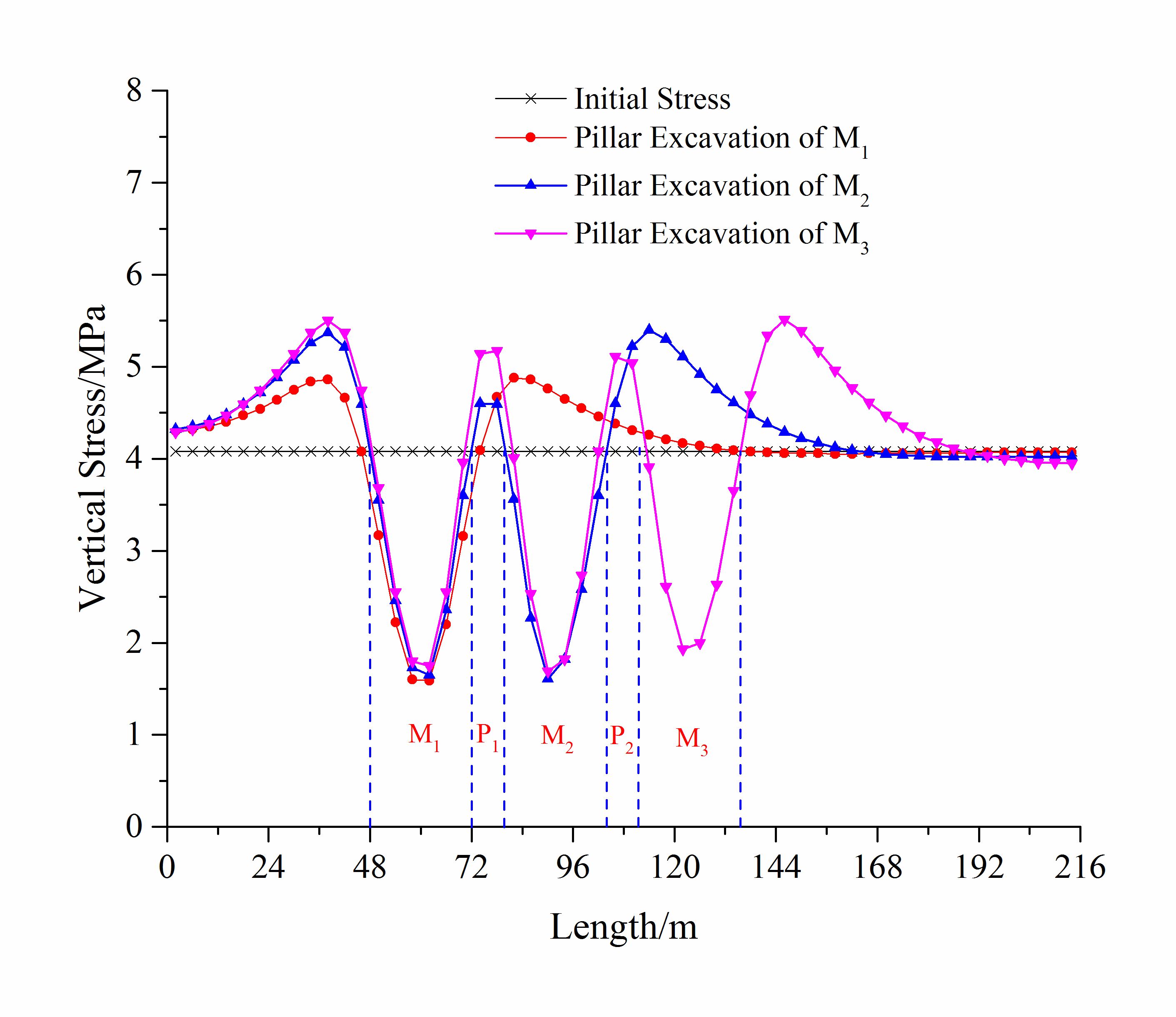  (**c**) | 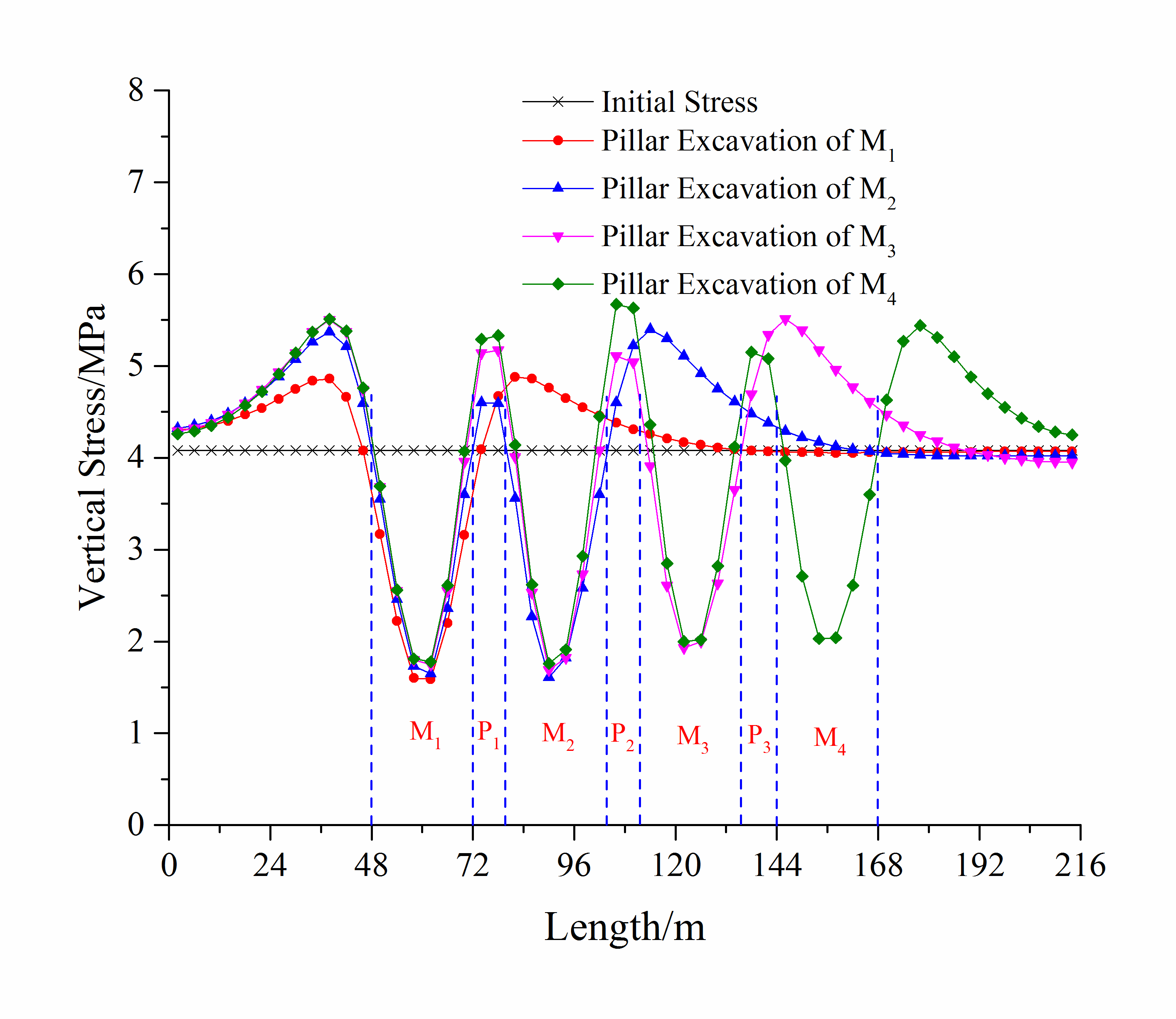  (**d**) |

**Figure 5.** Vertical stress evolution along strike length.(a) Vertical stress distribution after pillar excavation of M_1_, (b) Vertical stress distribution after pillar excavation of M_2_, (c) Vertical stress distribution after pillar excavation of M_3_ and (d) Vertical stress distribution after pillar excavation of M_4_.

| 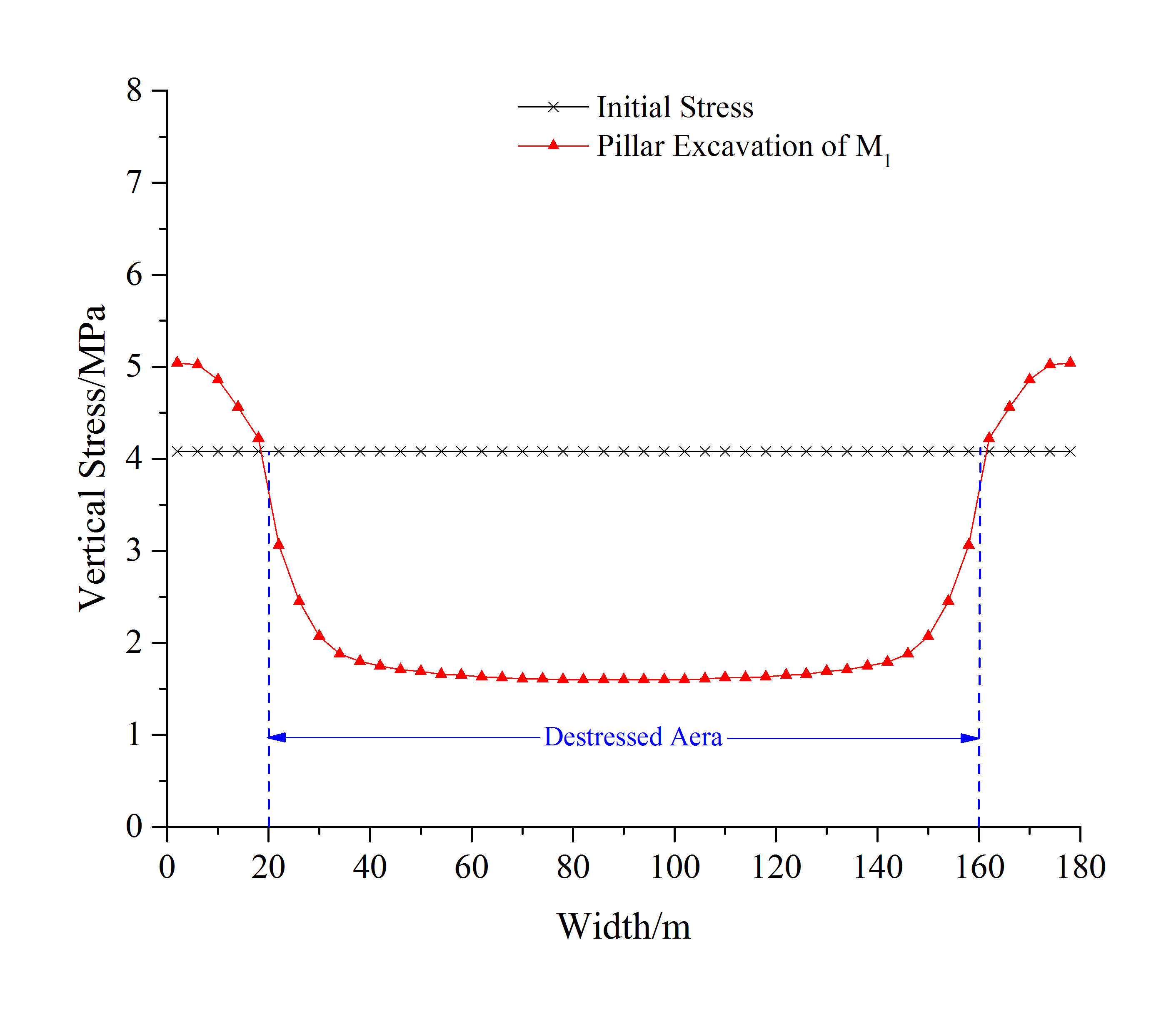 (**a**) | 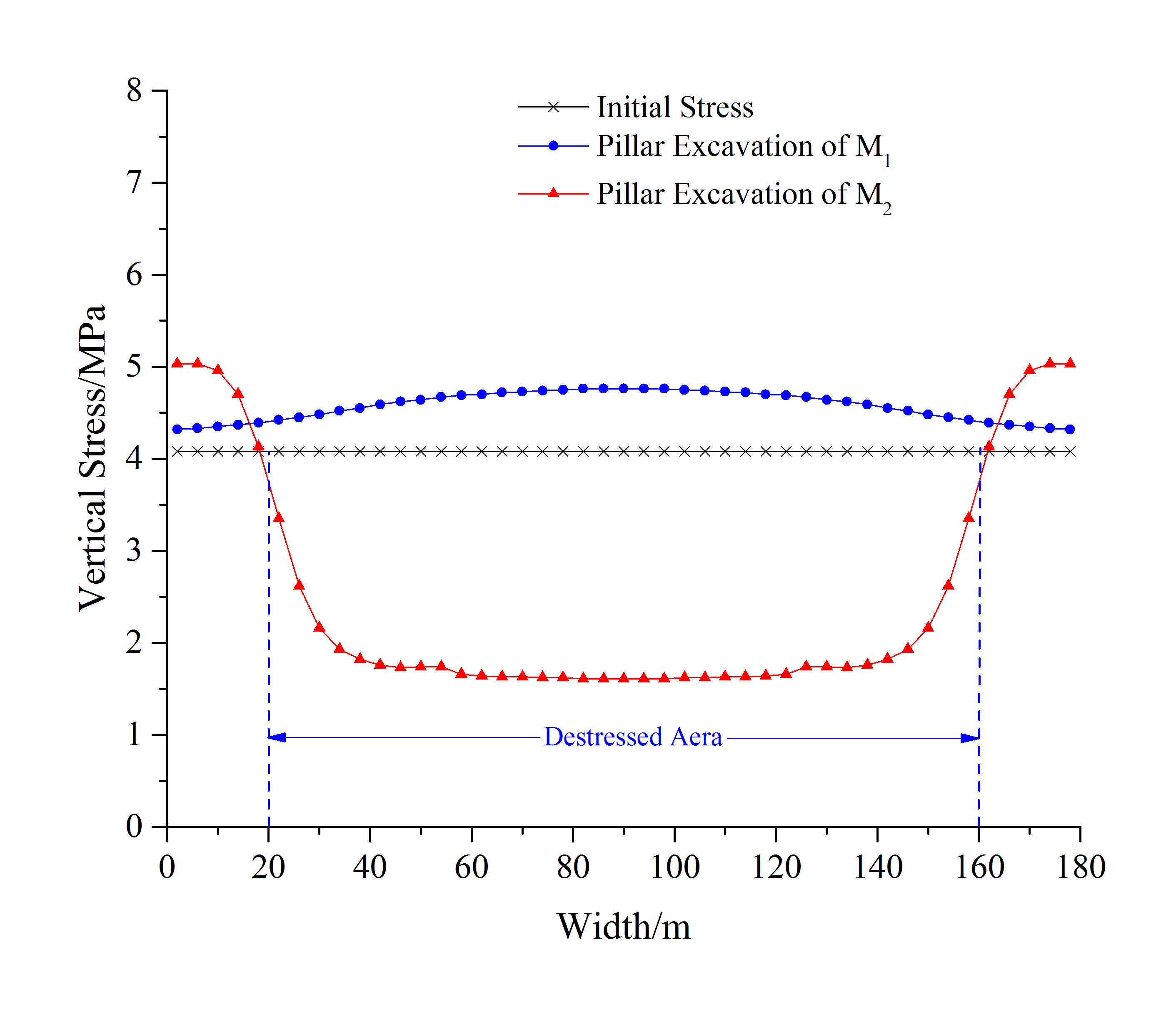  (**b**) |
| --- | --- |
| 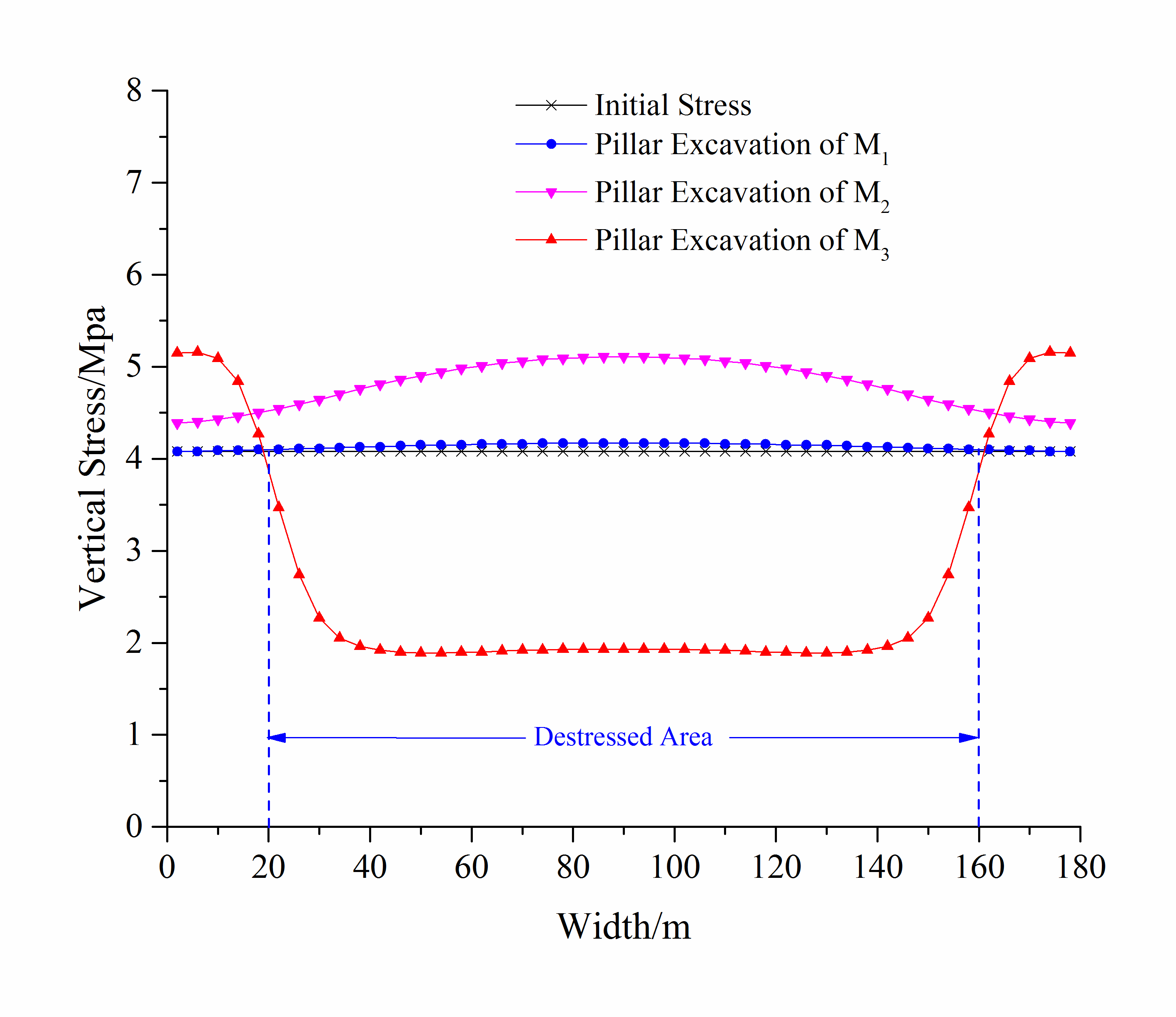  (**c**) | 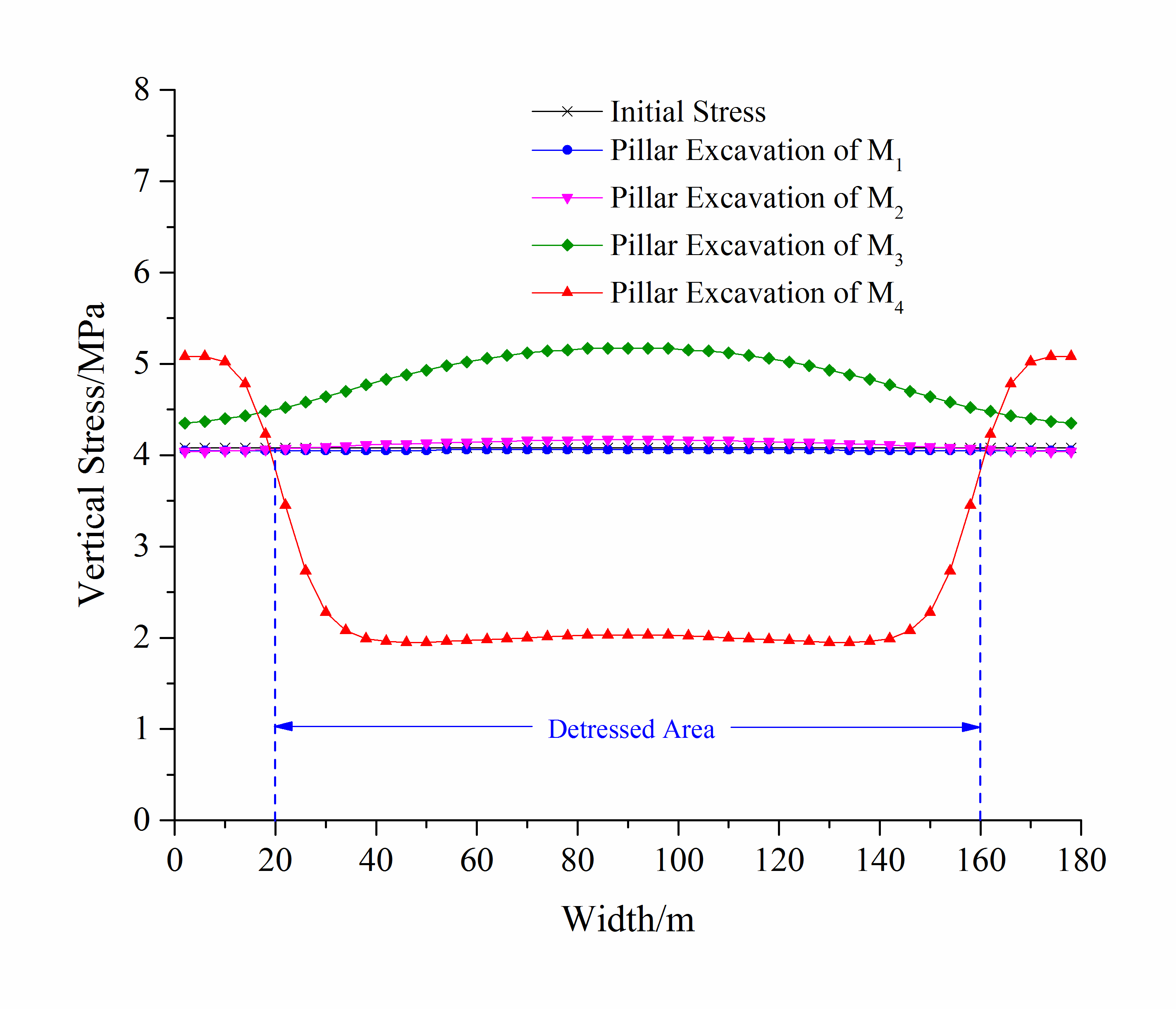  (**d**) |

**Figure 6.** Vertical stress evolution along dip width.(a) Vertical stress distribution along Line 2, (b) Vertical stress distribution along Line 3, (c) Vertical stress distribution along Line 4 and (d) Vertical stress distribution along Line 5.

| **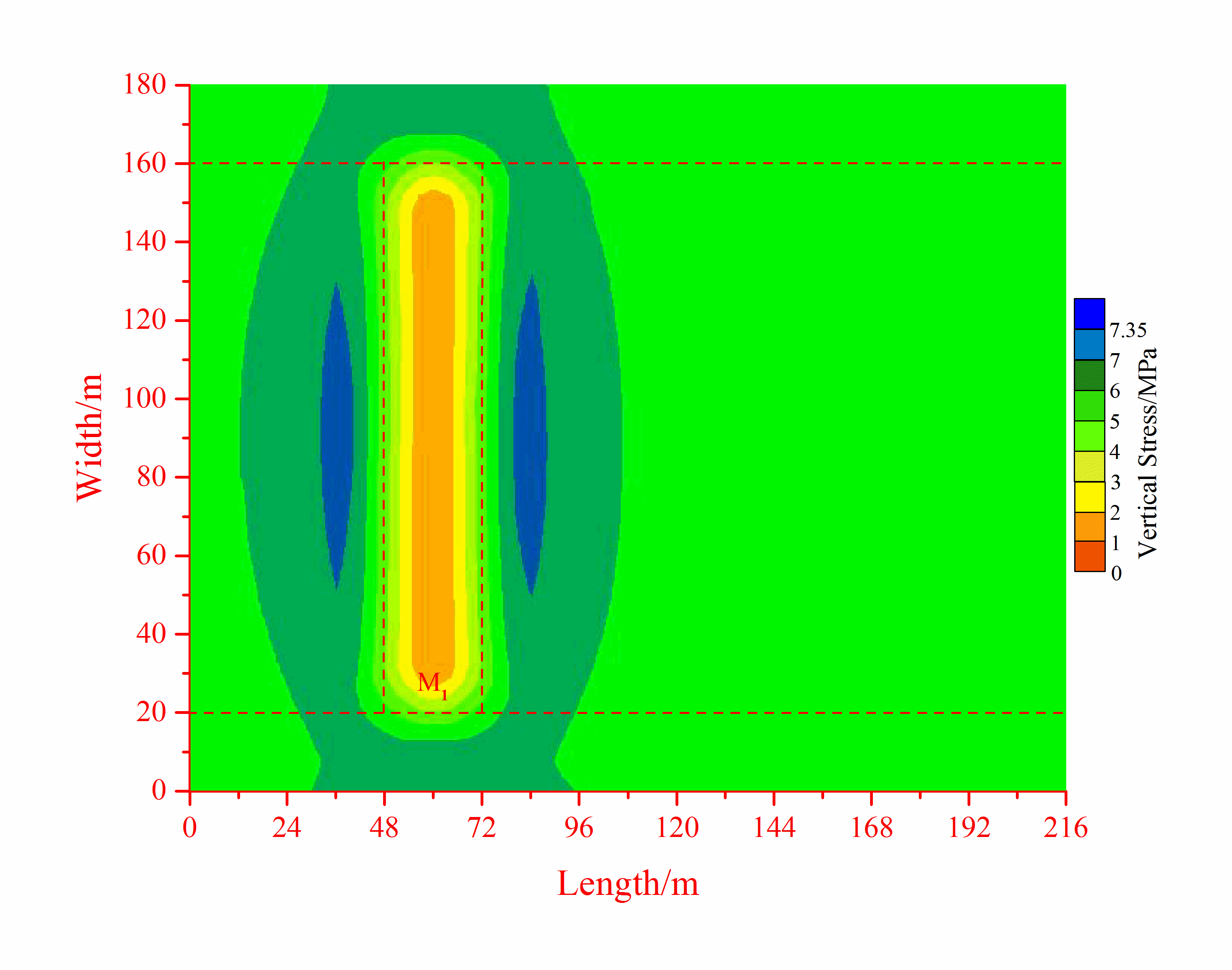** (**a**) | **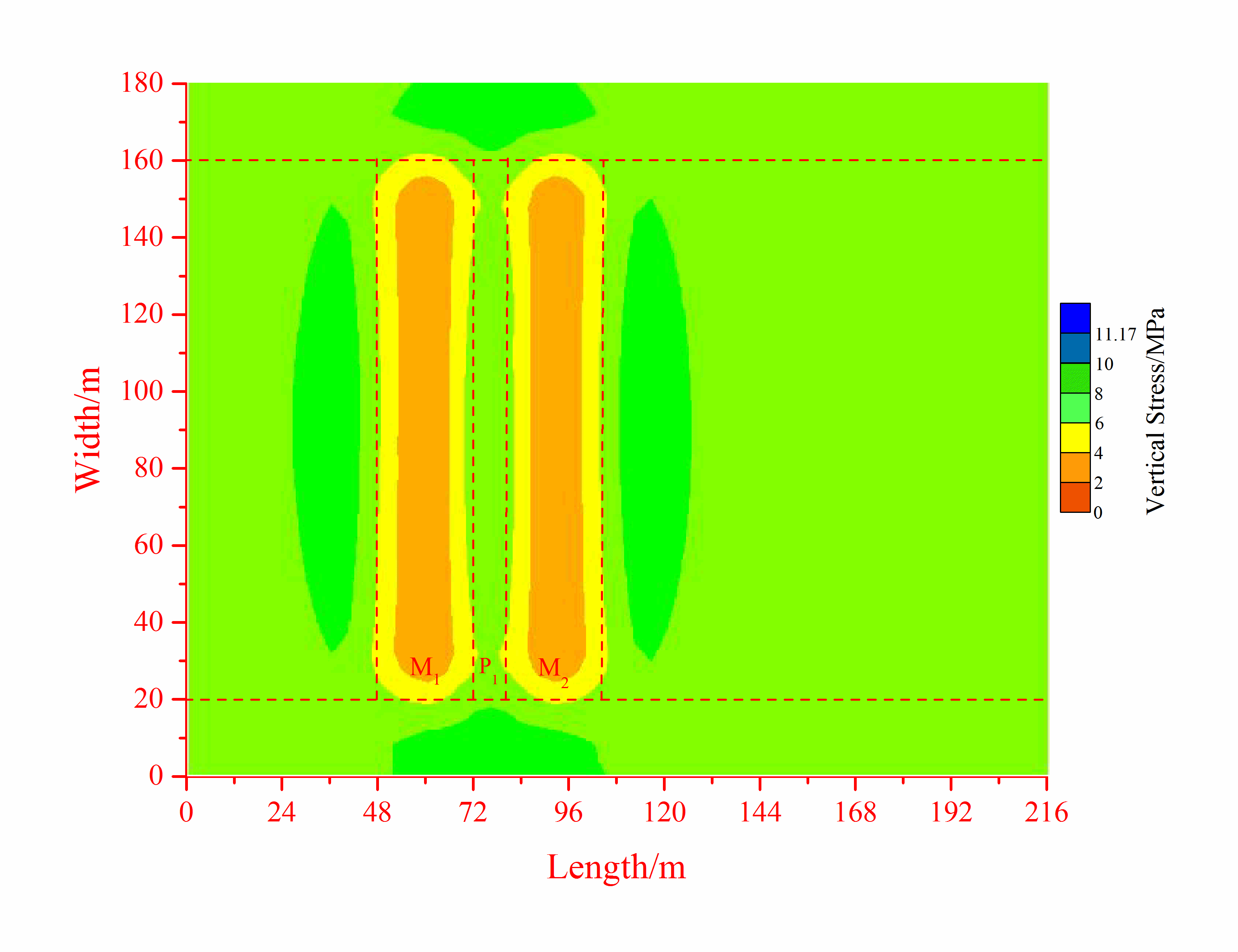**  (**b**) |
| --- | --- |
| **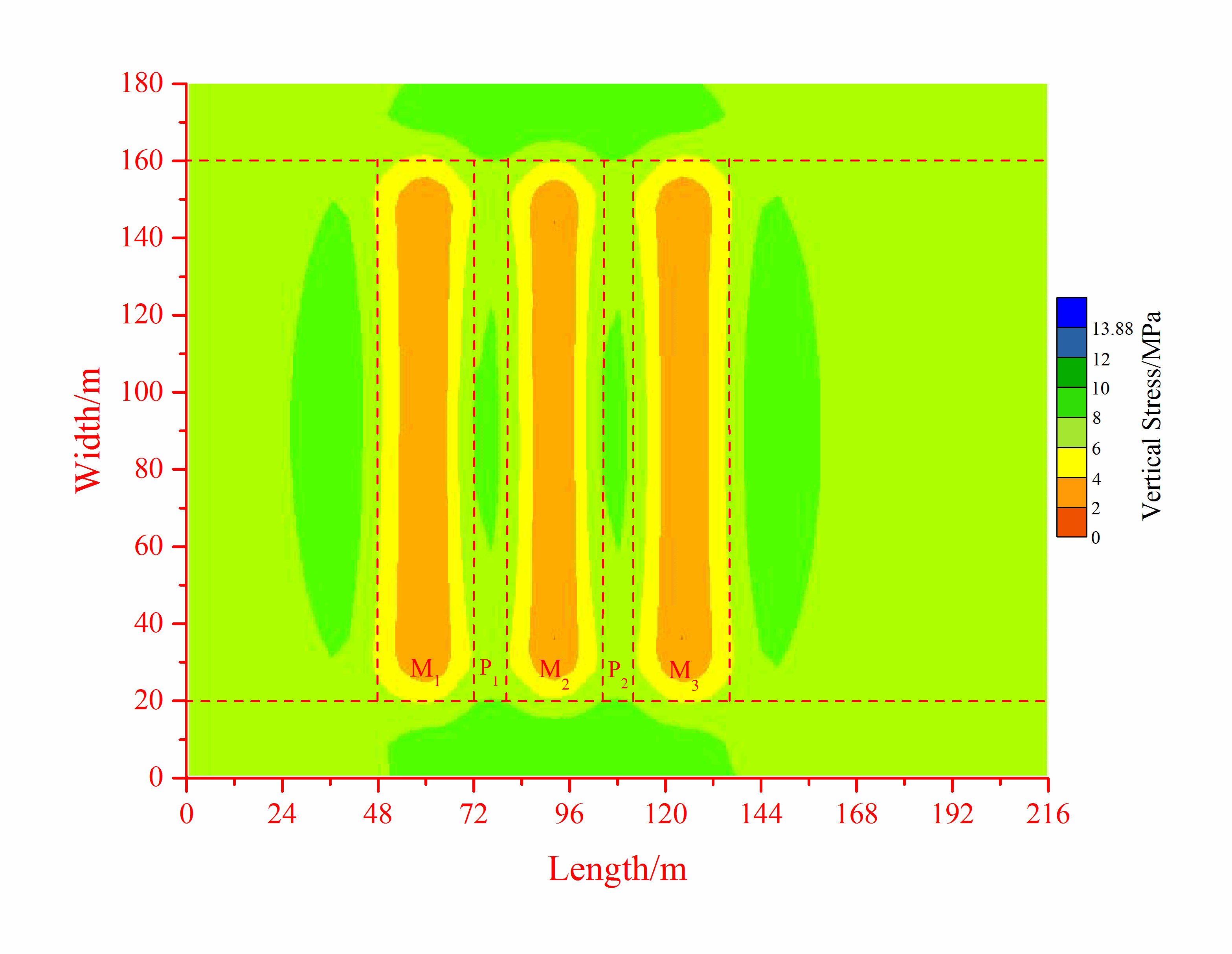** (**c**) | **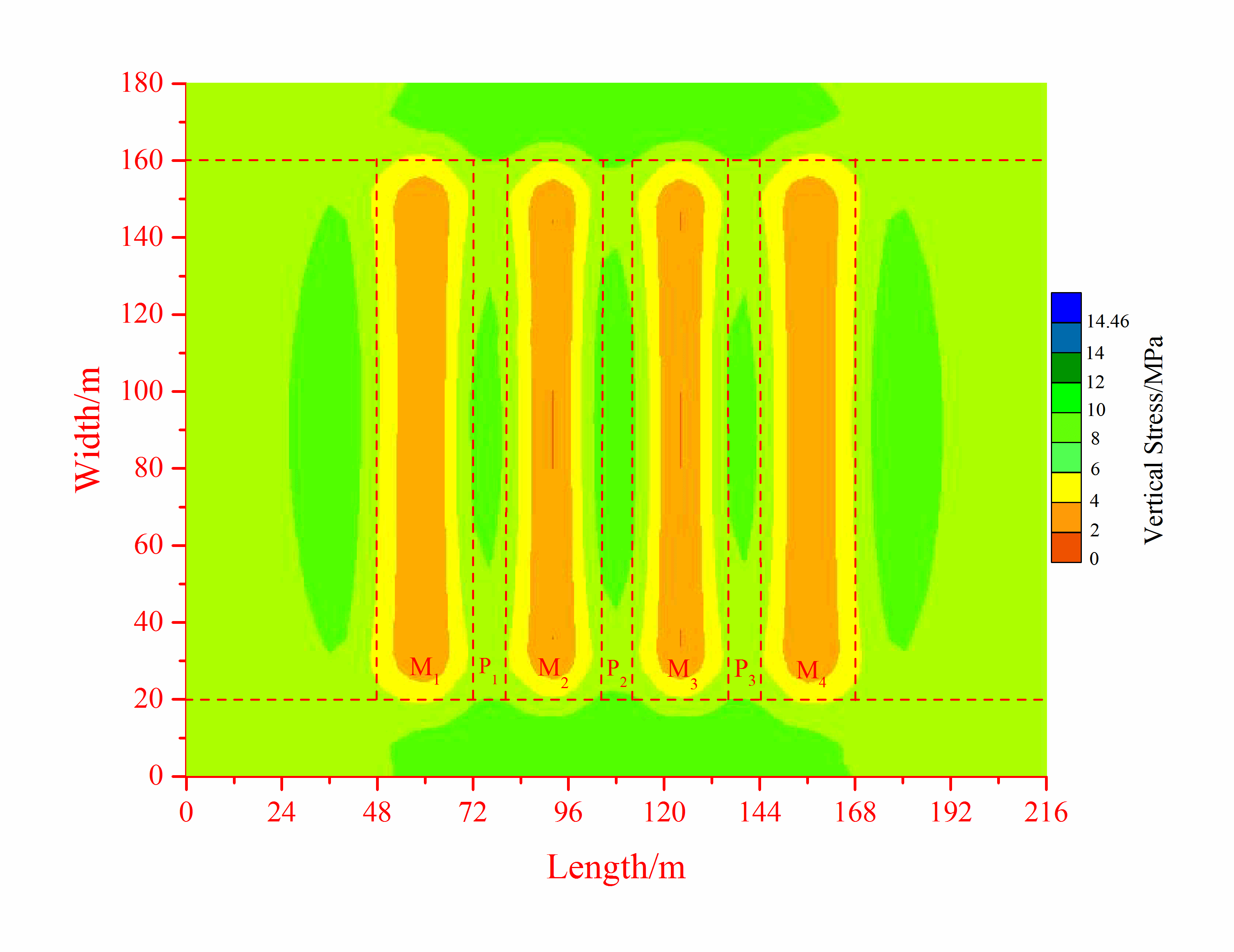**  (**d**) |

**Figure 7. Vertical stress distribution Contour after pillar excavation of coal seam No. 8.(a) after pillar excavation of M_1_, (b) after pillar excavation of M_2_, (c) after pillar excavation of M_3_ and (d) after pillar excavation of M_4_.**

| 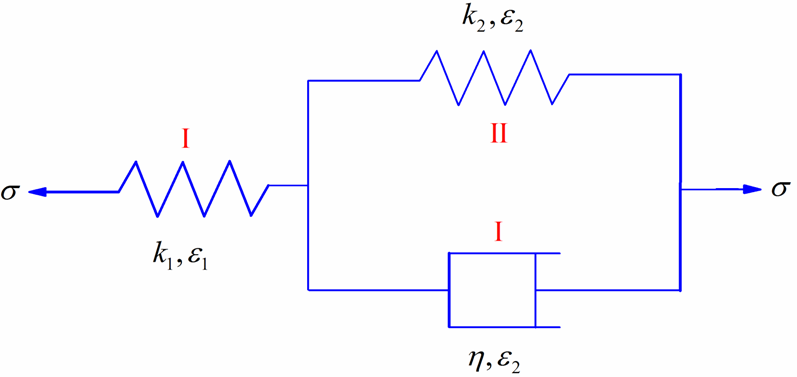 |
| --- |

**Figure 8. Mechanical model of a residual coal pillar.**

| 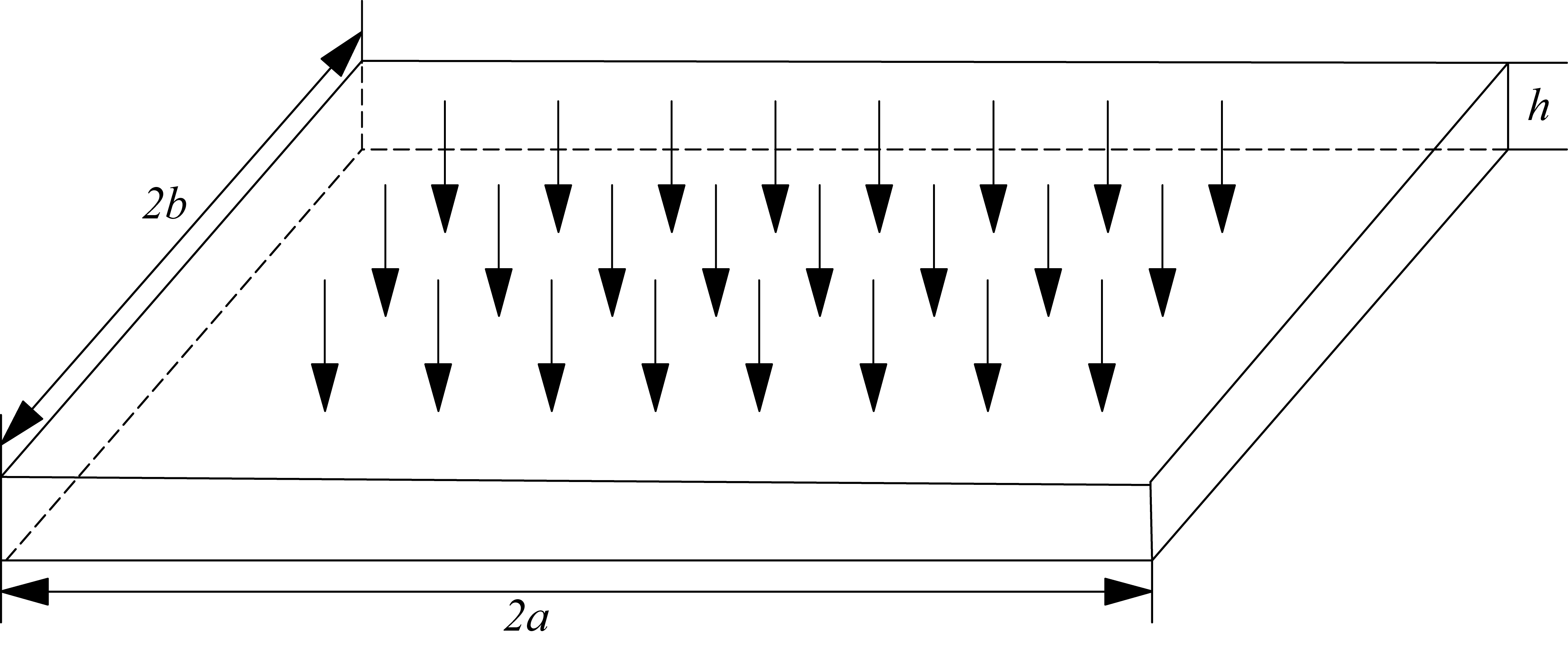  (**a**) | 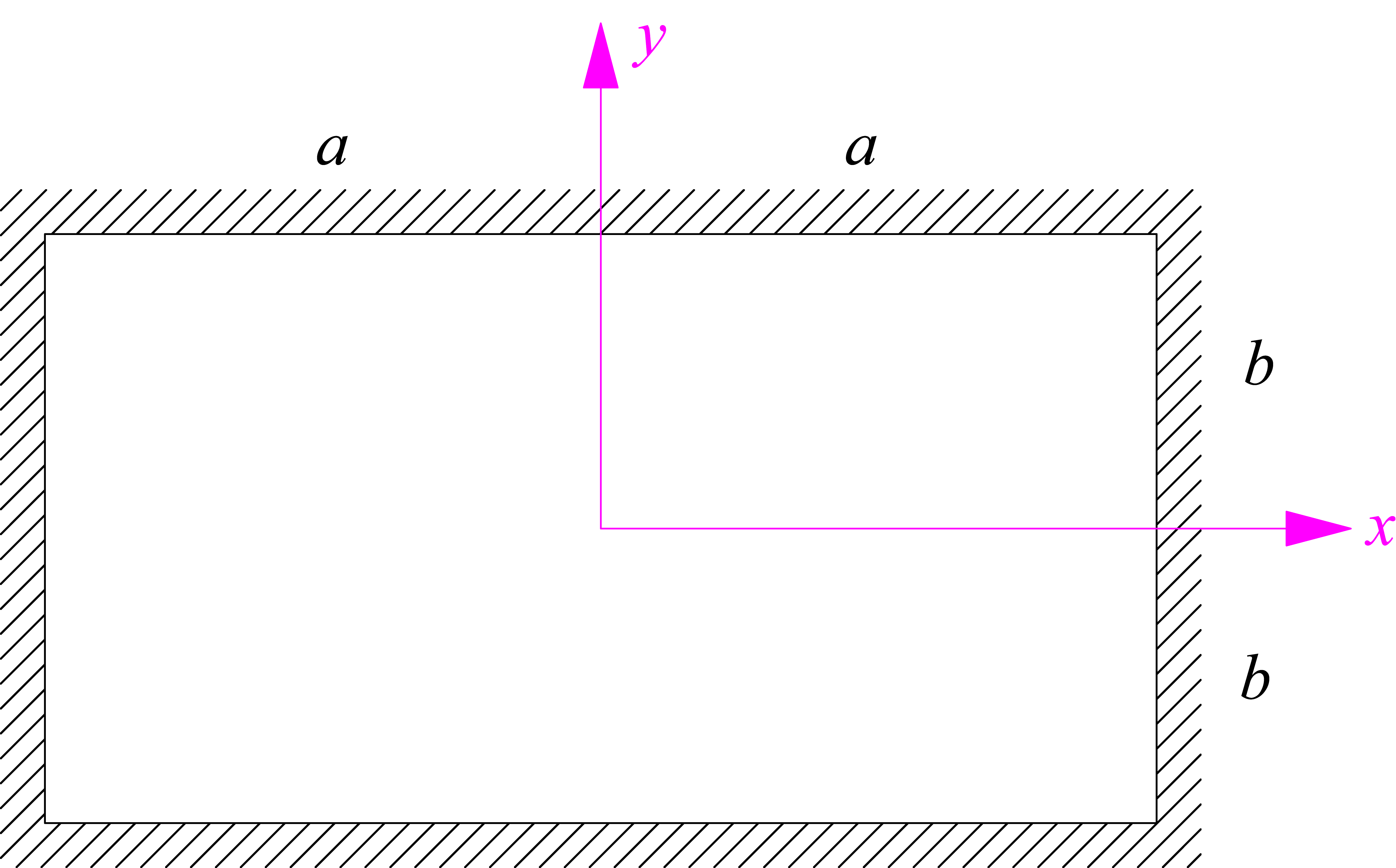  (**b**) |
| --- | --- |

**Figure 9.** Elastic rectangular plate with four fixed sides. **(a)** is the three-dimensional model of interburden; **(b)** is the two-dimensional model of interburden.

| 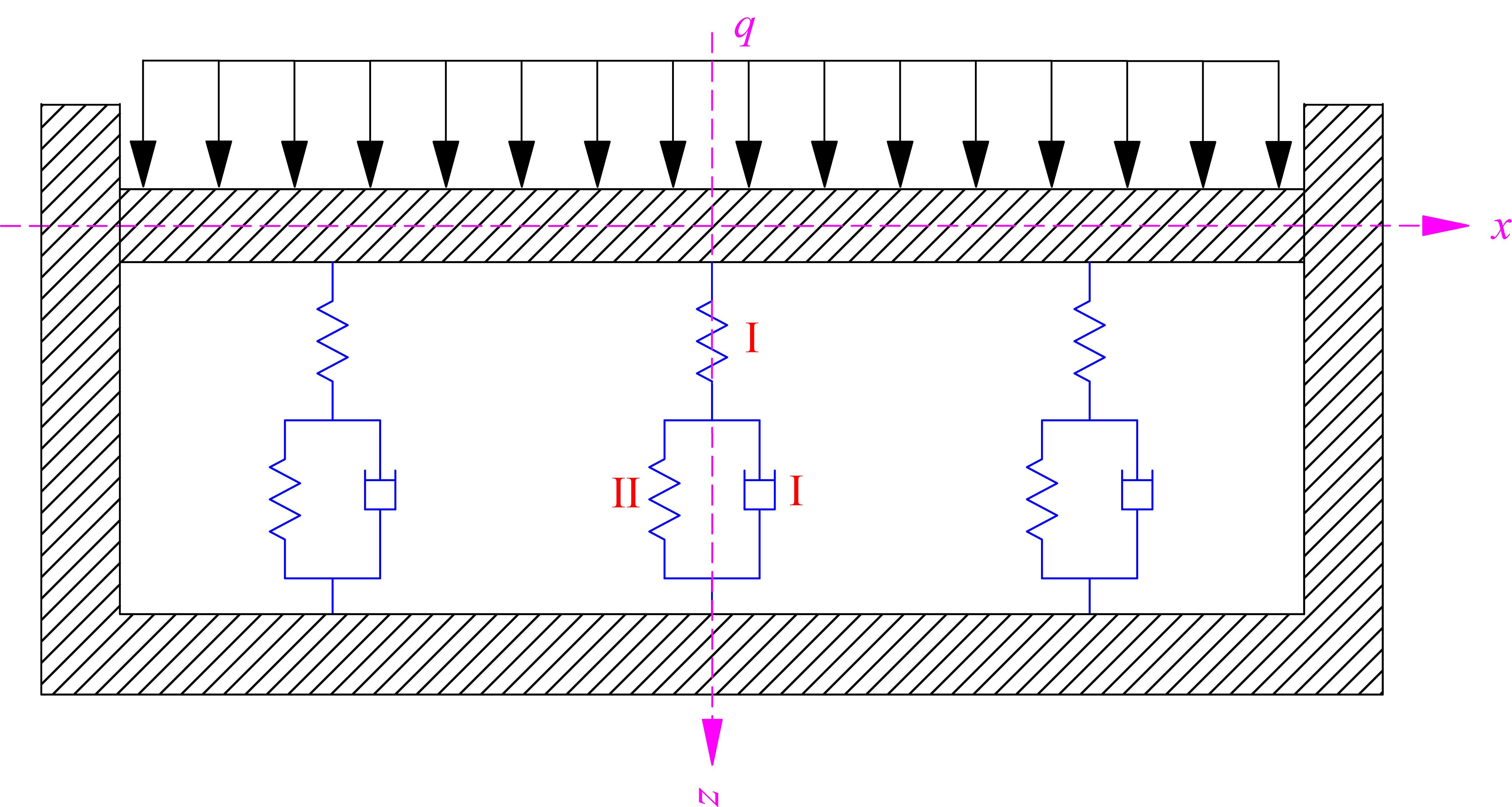 |
| --- |

**Figure 10.** Mechanical model of interburden over abandoned pillar working.

| 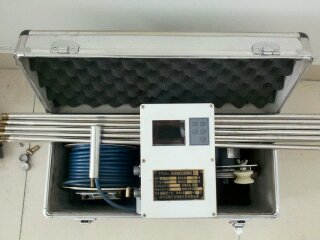 |
| --- |

**Figure 11.** TYGD10 high-definition borehole camera detection

| 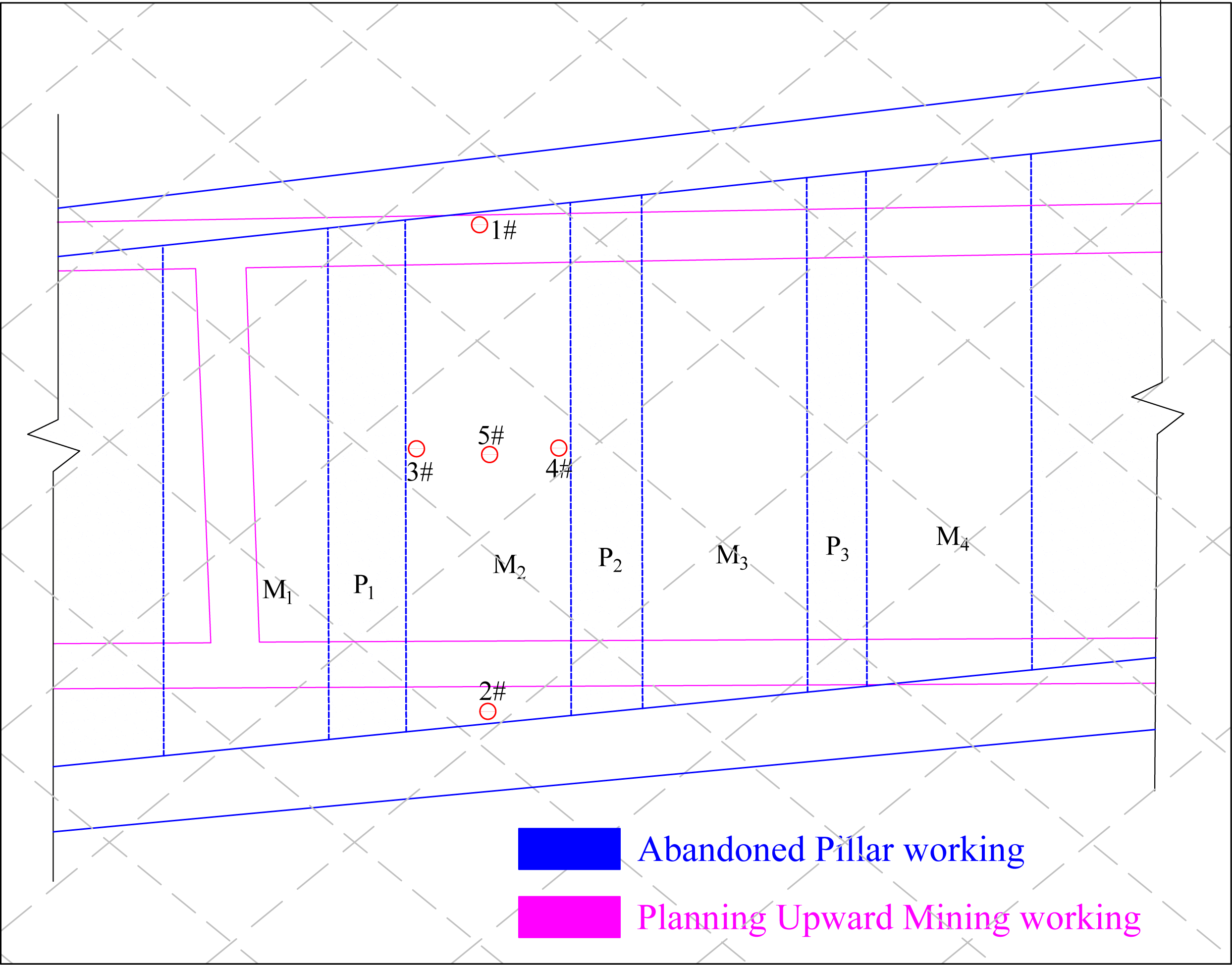 |
| --- |

**Figure 12.** Arrangement of monitoring boreholes

| 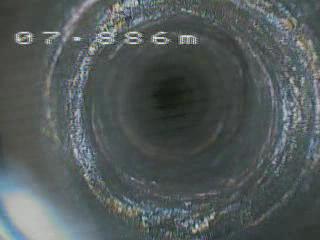 (**a**) | 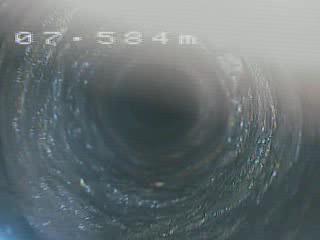  (**b**) |
| --- | --- |
| 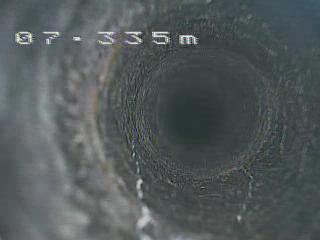  (**c**) | 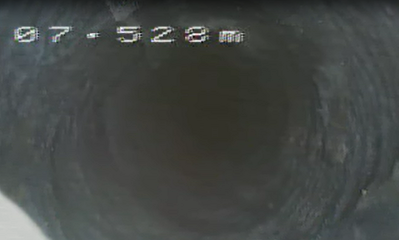  (**d**) |

**Figure 13.** Borehole images at boundary positions.(a) Boreholes 1#, (b) Boreholes 2#, (c) Boreholes 3# and (d) Boreholes 4#.

| 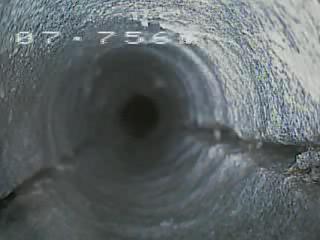 |
| --- |

**Figure 14.** Borehole 5# image at central positio
