# Supplementary material for: Vertical stress and stability of interburden over an abandoned pillar working before upward mining: a case study
Source: R Soc Open Sci. 2018 Aug 8;5(8):180346. doi: 10.1098/rsos.180346 (PMC6124054; doi:10.1098/rsos.180346)
Supplement: Jinwen Bai_Tables_ESM.doc [file rsos180346supp3.doc]

**Table 1. Geotechnical parameters of rock and coal in the model**

| Lithology | Density  (g/cm3) | Poisson's ratio | Elastic Modulus (GPa) | Bulk modulus  (GPa) | Shear modulus  (GPa) | Friction Angle  (°) | Cohesion  (MPa) | Tensile strength  (MPa) |
| --- | --- | --- | --- | --- | --- | --- | --- | --- |
| Sandstone | 2.61 | 0.14 | 1.95 | 0.90 | 0.86 | 40.9 | 5.54 | 2.6 |
| Limestone | 2.71 | 0.22 | 5.95 | 3.54 | 2.44 | 35.0 | 6.40 | 4.0 |
| Calcium shale | 2.72 | 0.18 | 4.86 | 2.53 | 2.06 | 41.5 | 6.61 | 2.1 |
| Shale | 2.16 | 0.22 | 0.72 | 0.43 | 0.30 | 33.2 | 2.12 | 0.9 |
| Carbonaceous shale | 2.52 | 0.13 | 1.54 | 0.69 | 0.68 | 39.8 | 2.77 | 1.1 |
| Coal seam No. 6 | 1.31 | 0.32 | 0.21 | 0.19 | 0.08 | 34.2 | 0.58 | 0.4 |
| Coal seam No. 7 | 1.34 | 0.32 | 0.27 | 0.25 | 0.10 | 30.8 | 0.67 | 0.4 |
| Coal seam No. 8 | 1.13 | 0.31 | 0.21 | 0.28 | 0.12 | 34.6 | 0.63 | 0.5 |
